# Equity of maternal and child health services in Afghanistan: a spatiotemporal analysis of national survey datasets

**DOI:** 10.1136/bmjgh-2024-018577

**Published:** 2025-07-10

**Authors:** Tim Groteclaes, Saifuddin Ahmed, Cauane Blumenberg, Aluisio J D Barros, Mickey Chopra, Nadia Akseer

**Affiliations:** 1 Department of International Health, Johns Hopkins Bloomberg School of Public Health, Baltimore, Maryland, USA; 2 Center for Pediatric and Adolescent Medicine, Department of Neonatology, University Hospital Heidelberg, Heidelberg, Germany; 3 Department of Population, Family and Reproductive Health, Johns Hopkins University Bloomberg School of Public Health, Baltimore, Maryland, USA; 4 International Center for Equity in Health, Universidade Federal de Pelotas, Pelotas, Brazil; 5 causale consultoria, Pelotas, Brazil; 6 World Bank, Washington, District of Columbia, USA; 7 Modern Scientist Global, St. Catharines, Ontario, Canada

**Keywords:** Delivery of Health Care, Geographic information systems, Child health, Maternal health, Health Services Accessibility

## Abstract

**Introduction:**

Afghanistan’s healthcare system faces geopolitical instability and inequities in maternal and child health (MCH) services, particularly associated with a temporary collapse in funding in 2021. We analysed coverage levels and spatiotemporal trends in sociodemographic inequalities in the country using data from the 2010/2011 and 2022/2023 Afghan Multiple Indicator Cluster Surveys.

**Methods:**

The study’s primary outcome was an adapted Composite Coverage Index (CCI) that combined seven essential MCH interventions with corresponding inequality measures, evaluated using Slope Indices of Inequality. These interventions included antenatal care, skilled birth attendance (SBA), Bacillus-Calmette-Guérin (BCG), diphtheria-pertussis-tetanus, and measles vaccination and treatment for suspected pneumonia and diarrhoea. Inequalities were analysed across wealth, education and urban/rural status at both national and provincial levels.

**Results:**

The analysis revealed persistent socioeconomic inequalities across all strata, with the most significant economic disparities observed in SBA and the largest educational disparities in vaccine coverage in 2022/2023. Nationally, the CCI increased by 4.2% from 42.1% (95% CI 40.3% to 44%) in 2010/2011 to 46.2% (95% CI 44.6% to 47.9%) in 2022/2023. Despite a slight increase between the two studies, severe regional disparities are masked, particularly in the eastern and southern regions, where coverage across multiple interventions significantly declined. The provinces of Nooristan and Urozgan significantly lost coverage, while Daykundi and Nimroz recorded increases in coverage and equity.

**Conclusion:**

The findings underscore the persistence of substantial inequalities in Afghanistan, with severe consequences for already vulnerable populations facing multiple hardships. The findings highlight ways in which geopolitical instability affects healthcare equity. Increasing disparities threaten to exacerbate existing challenges in accessing essential healthcare services, particularly for those of lower socioeconomic status. Urgent, targeted interventions are necessary to address these inequities, the impacts associated with funding cuts and gender marginalisation, and to mitigate their detrimental impact on Afghan women and children.

**WHAT IS ALREADY KNOWN ON THIS TOPIC**

- Afghanistan’s health system has struggled for decades, with high maternal and infant mortality rates despite some progress post-2001.
- A chaotic period, associated with the 2020 Doha Agreement, contributed to the Taliban’s return to power in 2021 and led to disruptions in healthcare services and funding.
- This has aggravated concerns for maternal and child health.
- Pre-existing (spatial) inequalities in healthcare access were substantial, particularly in rural areas.
- Reports and anecdotal evidence suggest a declining quality of care and decreased utilisation of healthcare services, but neither of these trends has been confirmed by large-scale studies.

**WHAT THIS STUDY ADDS**

- This provides the first large-scale analysis of maternal and child healthcare access and equity in Afghanistan after August 2021.
- Shifting trends in maternal and child health service coverage are highlighted, contributing new evidence on post-transition healthcare access in Afghanistan.
- For instance, trends reported here uncover growing economic inequalities in BCG vaccination and a reduction in educational disparities for skilled birth attendance.

**HOW THIS STUDY MIGHT AFFECT RESEARCH, PRACTICE OR POLICY**

- We provide a critical analysis of maternal and child health service coverage in Afghanistan to inform future research, health intervention and the work of international donors.
- Analyses reveal persistently low coverage among the most vulnerable groups, particularly in remote and socioeconomically disadvantaged areas.
- These data imply potential public health risks, including a resurgence of other preventable diseases like polio.
- This work emphasises the urgent need for coordinated humanitarian and development efforts to address health inequities.

## Introduction

 Afghanistan’s past and present are shaped by natural disasters, geopolitical instability, a fragile healthcare infrastructure and spatial as well as societal inequities in maternal and child health (MCH) services.^1^,^3^

While sociocultural practices like early marriage and the necessity for male permission influence maternal health across Central and Southern Asia,^4^ Afghanistan presents unique and sometimes counterintuitive patterns.^5^ Challenges are further exacerbated by the widespread exclusion of women from public life, denial of higher education and severe limitations on their freedom of movement.^6^ Additionally, under-age marriages and subsequent early childbearing have surged, especially in socioeconomically unstable environments.^7^

Before the Taliban came to power, Afghanistan experienced a noteworthy decline in maternal, newborn and child mortality rates between 2001 and 2020.^8^ However, despite this progress, the country was already not on track to meet the Sustainable Development Goals by 2030.^9^ In 2020, for example, Afghan mothers faced a lifetime risk of one maternal death in every 32 women, representing the eighth highest rate worldwide. The maternal mortality rate stood at 620 deaths per 100 000 live births.^8^

When compared with neighbouring countries, Afghanistan’s maternal health indicators follow a similar regional pattern but often with considerably greater severity.^8^ In the broader South and Central Asian region, the average maternal mortality rate was 129 deaths per 100 000 live births, and the lifetime risk of maternal death was one in 340 women in 2020.^8^ Although these figures remain high, they are substantially less severe than those observed in Afghanistan. Furthermore, it is notable that no other South or Central Asian country ranked among the 50 countries with the highest lifetime risk of maternal death.^8^

Despite the difference in intensity, Afghanistan’s challenges are not unique. Consistent with findings from other low- and middle-income countries (LMICs) in South and Central Asia, like Pakistan, India or Laos, female education and timely access to high-quality care were identified as key determinants of maternal health.^4^

With regard to neonatal health, Afghanistan, along with Pakistan and India, has made slower progress in reducing neonatal mortality rates compared with other South Asian countries. These three countries have not met their Millennium Development Goals for reducing neonatal mortality.^10^

Despite notable improvements over the last two decades, concerns about healthcare access increased during the transition period that began with the Doha Agreement on 29 February 2020. This agreement was a peace deal negotiated between the USA and the Taliban that established terms for the withdrawal of US troops from Afghanistan and set conditions for intra-Afghan negotiations.^11^ The situation saw a peak on 1 May 2021, with the start of the final withdrawal of US forces together with the start of the Taliban’s offensive. At this point, nearly 20% of the country was under Taliban control.^12 13^ Following the Taliban’s takeover in August 2021 and the associated collapse of international funding,^14 15^ the future of the healthcare system became uncertain, as it largely relied on the subsidised practice of contracting local non-governmental organisations (NGOs).^16^ Estimates projected an increase of 2 862 maternal, 15 741 neonatal and 30 519 child excess deaths concurrent with the pause in subsidies from the Afghanistan Resilience Trust Fund (ARTF) among others.^17^ The ARTF is a multidonor trust fund established in 2002 and administered by the World Bank to coordinate international assistance for Afghanistan.^18^

Afghanistan’s current challenges are comparable to other postconflict settings like the Democratic Republic of the Congo, Haiti or Sudan. Main issues in these cases were an uneven distribution of healthcare access, political instability and ongoing security concerns, and an understaffed healthcare system.^19^ In more detail, Afghanistan’s new healthcare landscape is said to be characterised by overworked staff, declining quality of care and decreasing utilisation of services.^20^ This is reflected by a one-third decrease in child immunisation coverage^21^ and the closure of almost 90% of essential healthcare facilities immediately after August 2021. Malnutrition spiked among children under five, disproportionately in girls.^22 23^ Furthermore, in 2023, 25% of the Afghan population lived in underserved areas, which are mainly located in rural and mountainous regions.^24^

Similar to the reported drop in immunisation coverage, Yemen saw a 24% reduction in vaccination rates after conflict escalated in 2015, while certain regions of South Sudan experienced declines of up to 29% during political transitions.^25^

Other studies from South Sudan and Syria link declines in key MCH indicators to barriers like financial constraints, inadequate or inaccessible infrastructure, lack of transport, security concerns and cultural norms.^26 27^

Literature and especially data analyses published after August 2021 are scarce. Most knowledge about the sociocultural and healthcare landscape is drawn from anecdotal reports. All currently available assessments use temporally and politically outdated pre-Taliban datasets like the 2010/2011 Afghan Multiple Indicator Cluster Surveys (MICS).^2^ While it is too early to draw definitive conclusions about the Taliban’s influence on healthcare access, the UNICEF 2022/2023 MICS provides the first large-scale survey and in-depth insight into the healthcare system since the start of the transition period that began with the Doha Agreement and the associated regime change.^28^ This presents a unique opportunity to analyse and compare two standardised datasets, allowing for the examination of spatial and temporal changes in healthcare access.

To provide policymakers with essential insights into the current state of healthcare in Afghanistan, this study aims to investigate the temporal trends (2010/2011 vs 2022/2023) and geographical disparities (across all 34 Afghan provinces) in healthcare access and equity. Specifically, the study assesses inequalities related to wealth, urban/rural residence and maternal education and examines how these disparities intersect over time and across regions. By analysing these factors, the study seeks to offer a nuanced understanding of the healthcare landscape and identify subpopulations and geographical areas that are most in need of targeted policy and programmatic interventions.

## Methods

### Study setting

Afghanistan is a diverse nation comprising about 40 million people with various ethnicities.^29^ The country’s geography ranges from rugged mountains to arid deserts,^24^ with a significant portion of its population residing in rural areas (74.6% in 2022/2023).^28^ Afghanistan is ethnically divided, reflecting a complex social structure shaped by its turbulent history. Its society is composed of at least 24 different ethnic groups, with the majority being Pashtun (40%) or Tajik (39%).^30^ Amidst ongoing conflict, the country’s demographics also highlight a predominantly young population, with a median age of about 18 years and a fertility rate of around 4.3 live births per woman.^29^

Spatiotemporal trends were studied at the country level, the geographical region and the province level. The geographical units used for this analysis are the 34 Afghan provinces, as well as the eight geographical regions used by UNICEF in the MICS report from 2010/2011.^31^ The regions include Central, Central Highlands, East, North, North East, South, South East and West (online supplemental figure 1).

### Study design

This study is a comparative analysis of two cross-sectional surveys conducted about 12 years apart (2010/2011 and 2022/2023), aimed at assessing MCH indicators across Afghanistan by province and region. The strong similarity in survey design and methodology allows for a robust and valid comparison of the findings.

### Data source

Sources are the 2010/2011 and the 2022/2023 MICS household survey data from Afghanistan.^28 31^ MICS is a large-scale, interview-based, nationally representative survey conducted using a multistage, stratified cluster sampling method that focuses on the well-being of children and women.^28^ The datasets are publicly available through the UNICEF MICS repository at https://mics.unicef.org/, following a short registration process. The data are free to use for non-commercial purposes under UNICEF’s terms of use, with no embargo or licence needed. A total of 13 468 households, 22 053 women (aged 15–49) and 15 327 children (<5 years) were sampled in the 2010/2011 survey, and 23 338 households, 44 874 women (aged 15–49) and 33 398 children (<5 years) were sampled in the 2022/2023 survey. Response rates ranged from 95.1% to 98.8% depending on the subpopulations.^28 31^

While the structure of both surveys remained the same, the 2022/2023 survey was conducted under Taliban governance and restrictive policies on women’s rights. In contrast to 2010/2011, the 2022/2023 study omitted all contraceptive-related questions present in earlier surveys. Despite their shared methodological framework, contextual differences in governance and content partially affect the comparability of the two surveys.^28 31^

### Indicator selection

The selection of indicators was informed by previous publications using the Composite Coverage Index (CCI) and the Countdown definitions.^32 33^ The CCI was originally invented to provide a comprehensive measure for progress and inequalities in reproductive, maternal, newborn and child healthcare. The indicators were selected to provide a balanced overview of curative and preventive MCH services, including immunisation coverage and community-level healthcare-seeking behaviour.^34^ The individual indicators that comprise the CCI were additionally selected based on their abundant availability throughout different datasets and surveys, a strong correlation with under-five and neonatal mortality, and the fact that the CCI is less influenced by sampling variability compared with the individual indicators’ variability.^34 35^

Based on the available data and following the revised CCI, we used seven indicators. First, the proportion of four or more antenatal care (ANC) visits among all women who had a live birth in the past 2 years (ANC4). Second, skilled birth attendance (SBA), defined as any live birth occurring in the last 2 years attended by at least a doctor, a nurse or an (auxiliary) midwife. The third, fourth and fifth indicators were related to immunisation: coverage of children between 12 and 23 months with BCG vaccination, three doses of at least a tetravalent diphtheria-pertussis-tetanus (DPT3) vaccination and the first dose of the measles (MSL) vaccination schedule. The sixth indicator assessed care seeking with a skilled provider for children under the age of 5 who had suspected pneumonia (or acute respiratory illness - ARI), and the seventh indicator looked at the treatment of diarrhoeal diseases with oral rehydration solution or salts (ORS) and continued feeding among children under five (online supplemental table 1). Data for the commonly used eighth indicator, the demand for modern contraceptive methods, were not available in the MICS 2022/2023 survey.

### Composite Coverage Index

Dropping the contraceptive variable, we used a modified version of the revised CCI.^36^ The 2010/2011 dataset was reanalysed to ensure comparability of the indices. Confidence intervals (CIs) for the CCI were calculated via jackknifing at the cluster level. This is a leave-one-out method where CIs are calculated by recalculating the CCI multiple times, leaving out one cluster per calculation until each cluster has been omitted exactly once (online supplemental appendix page 2).


$$CCI=\frac{\frac{ANC4+SBA}{2}+\frac{BCG+MSL+2\cdot DPT3}{4}+\frac{ORS+ARI}{2}}{3}$$

### Equity dimensions

To assess socioeconomic inequalities, we defined three socioeconomic equity dimensions: maternal education level, wealth (grouped in quintiles) and urban or rural residence (online supplemental appendix page 2).

### Statistical analysis

Sampling weights provided with the MICS datasets were used throughout the analysis (online supplemental appendix page 2). For visual display of the stratified indicators and the CCIs, we plotted the individual values as *equiplots* for each equity dimension. The 2010/2011 study used regional groups (online supplemental figure 1) as the main geographical strata. To ensure comparability and representativeness, we regrouped the 2022/2023 survey data, originally stratified by provinces, into these higher level regional groups. This regrouping helped achieve consistent sample sizes across strata using an arbitrary threshold of 30 individuals per stratum but simultaneously led to a reduced spatial resolution.

### Slope Index of Inequality

To quantify inequalities, we used two different approaches. As a simplistic method, we calculated the crude mathematical differences between the highest and the lowest strata as displayed in online supplemental table 2. To further specify the extent of absolute inequality, we calculated the Slope Index of Inequality (SII) for each intervention and both economic and educational stratification. Based on a logistic regression of all groups within a stratum, the SII displays the absolute inequality as a percentage point (pp) difference between the highest and lowest ends of the equity dimension of interest. For example, the SII displays the difference between the richest and the poorest 20% of the population in absolute percentage points while still considering the distribution among the remaining 60% of the people. Calculations with non-linear correlations between coverage and strata were omitted. A computation of the SII for the urban/rural dimension was not possible due to the binary and non-ordinary character of the variable. We, therefore, calculated the crude differences between urban and rural coverage and the corresponding CIs through linear regression and a linear combination.

### Temporal comparison

To provide a temporal comparison of the development of inequalities, we calculated the percentage point difference in SII for each intervention and equity dimension. A non-overlapping 95% CI of the SIIs from both time points for the same intervention and strata was considered significant.

In the 2022/2023 dataset, all children (100%) aged 12–23 months were born after the Doha Agreement, that is, after the onset of the political transition phase. Of these, about two-thirds (62.9%, or 4014 children) were born on or after 1 May 2021, which marked the beginning of the Taliban’s offensive coinciding with the start of the final withdrawal of US troops, associated with a peak in violence and instability.

Comparing CCIs across the two studies using the standard definitions displayed in online supplemental table 1 entails the inclusion of some children in the 2022/2023 dataset who were born before 1 May 2021. To ensure methodological consistency, we adhered to these standard definitions for most analyses. However, all indicators of the 2022/2023 survey were assessed after the signing of the Doha Agreement and thereby reflect a time point during this phase of political change.

To specifically examine the influence of the most intense phase of this period following 1 May 2021, and the growing impact of the Taliban, we conducted an additional analysis that diverged from strict comparability. In this analysis, we included only births and pregnancies after 1 May 2021 within the 2022/2023 dataset to depict and compare the status quo after these significant events.

All statistical analyses were done with STATA/BE V.18.0, partially using the publicly available *equiplot.ado* and *siilogit.ado* packages.^37^ The maps were created in ArcGIS Pro. A p value below 0.05 (two sided) was considered statistically significant. Missing data were handled using complete case analysis. All aspects related to patient and public involvement were the responsibility of UNICEF and its partners. Patients and the public were not involved in any way in this study.

## Results

### Population composition

We examined data from 14 871 children under the age of 5 from 2010/2011 and 32 989 children under the age of 5 from 2022/2023, along with data from 21 290 women of reproductive age from 2010/2011 and 44 340 from 2022/2023 (table 1).

**Table 1:** Demographics of both study populations as weighted numbers and corresponding proportions

	MICS 2010/2011	MICS 2022/2023	Total
Households	13 116 (100%)	23 213 (100%)	36 329
Urban	2427 (18.5%)	4107 (27.3%)	
Rural	10 689 (81.5%)	19 106 (72.7%)	
Women aged 15–49 years	21 290 (100%)	44 340 (100%)	65 630
Live birth in the past 2 years*	4865 (35.7%)	12 507 (28.2%)	
Birth on or after 29 February 2020†	N/A	12 507 (100%)	
Birth on or after 1 May 2021‡	N/A	10 233 (81.2%)	
Birth on or after 15 August 2021§	N/A	8524 (68.2%)	
Female education*			
No or pre primary education*	17 359 (81.5%)	29 926 (67.5%)	
Primary education*	1595 (7,5%)	5180 (11.7%)	
Secondary+ education*	2330 (11%)	9233 (20.8%)	
Children aged <5 years	14 871 (100%)	32 989 (100%)	47 860
Children aged 12–23 months¶	2497 (16.8%)	6383 (19.4%)	
Born on or after 29 February 2020†	N/A	6383 (100%)	
Born on or after 1 May 2021‡	N/A	4014 (62.9%)	
Born on or after 15 August 2021§	N/A	2120 (33.2%)	
Maternal education**			
Mothers with no or pre primary education**	13 532 (91%)	25 993 (78.8%)	
Mothers with primary education**	698 (4.7%)	3119 (9.5%)	
Mothers with secondary+ education**	634 (4.3%)	3877 (11.8%)	
Diseased in the past 2 weeks			
Suspected pneumonia in the past 2 weeks††	2762 (18.6%)	8.550 (25.92%)	
Diarrhoea in the past 2 weeks††	3403 (22.9%)	12 593 (38.17%)	

Proportions not adding up to exactly 100% due to rounding error and missing data.

* Proportion among total number of women of reproductive age (15–49 years).

† Signing of the Doha Agreement initiating the transition period and withdrawal of international forces. Proportion among total number of women with a live birth in the past 2 years or total number of children aged 12–23 months, respectively.

‡ Date of the start of the Taliban’s offensive. Proportion among total number of women with a live birth in the past 2 years or total number of children aged 12–23 months, respectively.

§ Date of the political takeover of the Taliban. Proportion among total number of women with a live birth in the past 2 years or total number of children aged 12–23 months, respectively.

¶ Proportion among total number of children under 5 years.

** Proportion among mothers of all children under 5 years.

†† Proportion among all children under 5 years.

MICS, Afghan Multiple Indicator Cluster Surveys.

### Socioeconomic inequalities

#### Gap analysis

Table 2 displays key intervention coverage by equity dimensions for 2010/2011 compared with 2022/2023. In 2022/2023, the coverage was lowest for ANC4 (33.4%, 95% CI 31.9% to 35%) and ORS (21.7%, 95% CI 20.1% to 23.4%). All indicators showed a significant pro rich inequality pattern in 2022/2023 with higher coverage for the richest as compared with the poorest. The absolute economic inequalities were smallest for the two care-seeking interventions: ARI (SII 20.5 pp, 95% CI 13.3 to 27.8, p<0.001) and ORS (SII 13.5 pp, 95% CI 7.6 to 19.5, p<0.001). This indicates that the linearised absolute difference in coverage between the poorest and the richest quintile is 20.5 or 13.5 pp, respectively. Like in the survey from 2010/2011, the largest absolute economic inequality was found among SBA with almost 57 pp difference (95% CI 53.0 to 60.8, p<0.001) in coverage between the richest and the poorest.

**Table 2:** Coverage as proportions of each individual indicator for both surveys

Intervention	2010/2011				2022/2023				2022/2023 vs 2010/2011
	Total coverage% (95% CI)	Q1%(95% CI)*	Q5%(95% CI)†	SII (pp)(95% CI, P value)‡	Total coverage% (95% CI)	Q1%(95% CI)*	Q5%(95% CI)†	SII (pp)(95% CI, P value)‡	SII difference (pp)CI overlap (Y/N)§
ANC4	14.6(13 to 16.3)	5.8(3.9 to 8.6)	32.4(28.9 to 36)	29.6(24.6 to 34.1, <0.001)	33.4(31.9 to 35)	18.5(16.2 to 21)	52(48.2 to 55.8)	36.9(32.1 to 41.7, <0.001)	7.3 (Y)
SBA	38.7(35.8 to 41.6)	15.6(12.4 to 19.4)	76.3(72.9 to 79.4)	63.2(57.8 to 68.5, <0.001)	67.5(65.8 to 69.3)	42.7(39.4 to 46)	93.4(91.4 to 95)	56.9(53 to 60.8, <0.001)	−6.3 (Y)
BCG	64.2(60.4 to 67.7)	54.4(46 to 62.5)	79.3(74.6 to 83.3)	24.9(13 to 36.8, <0.001)	64.7(62.4 to 67)	45(39.8 to 50.4)	85.9(82.8 to 88.6)	43.6(37.3 to 50, <0.001)	18.8 (N)
DPT3	40.2(36.8 to 43.7)	28.9(22.5 to 36.2)	54.4(49.4 to 59.2)	25.2(14.7 to 35.6, <0.001)	51.3(48.9 to 53.8)	35(30.3 to 40)	70.5(65.9 to 74.7)	37.2(29.9 to 44.6, <0.001)	12.1 (Y)
MSL	55.5(51.7 to 59.1)	43.7(35.3 to 52.5)	68.1(63.1 to 72.7)	24.7(12.8 to 36.6, <0.001)	51.2(49 to 53.3)	40(35.6 to 44.5)	65.6(61 to 69.8)	27.8(20.7 to 35, <0.001)	3.1 (Y)
ARI	60.5(57.2 to 63.7)	46.4(39.5 to 53.3)	65.7(59.9 to 71.2)	19.1(9.5 to 28.8, <0.001)	45.4(43.2 to 47.5)	36.2(32.8 to 39.7)	55.7(48.8 to 62.5)	20.5(13.3 to 27.8, <0.001)	1.4 (Y)
ORS	39(34.3 to 44)	39.6(32.5 to 47.2)	35.6(30.9 to 40.7)	Non-linear¶	21.7(20.1 to 23.4)	16(13.7 to 18.6)	28.6(23.7 to 34)	13.5(7.6 to 19.5, <0.001)	N/A

Intervention	2010/2011				2022/2023				2022/2023 vs 2010/2011
	Total coverage% (95% CI)	EDU1% (95% CI)*	EDU3%(95% CI)†	SII (pp)(95% CI, P value)‡	Total coverage% (95% CI)	EDU1% (95% CI)*	EDU3%(95% CI)†	SII (pp)(95% CI, P value)‡	SII difference (pp)CI overlap (Y/N)§
ANC4	14.6(13 to 16.3)	11.8(10.4 to 13.3)	45.4(39.1 to 52)	39.5(33.6 to 45.4, <0.001)	33.4(31.9 to 35)	28.4(26.9 to 30)	55.4(51.5 to 59.3)	37(31.6 to 42.3, <0.001)	−2.5 (Y)
SBA	38.7(35.8 to 41.6)	34.2(31.4 to 37.2)	83(77.7 to 87.2)	66.2(59.6 to 72.8, <0.001)	67.5(65.8 to 69.3)	62.5(60.5 to 64.4)	88.5(85.9 to 90.6)	44.2(37.8 to 50.6, <0.001)	−22 (N)
BCG	64.2(60.4 to 67.7)	62.4(58.4 to 66.2)	85.9(77.8 to 91.4)	40.8(27.7 to 53.8, <0.001)	64.7(62.4 to 67)	58.5(55.8 to 61.1)	86.1(82.2 to 89.3)	52.7(45.1 to 60.3, <0.001)	12 (Y)
DPT3	40.2(36.8 to 43.7)	38.3(34.6 to 41.9)	64(54.9 to 72.2)	38.3(26.2 to 50.5, <0.001)	51.3(48.9 to 53.8)	44.4(41.7 to 47.1)	74(69 to 78.3)	52.7(45.5 to 59.9, <0.001)	14.4 (Y)
MSL	55.5(51.7 to 59.1)	53.5(49.5 to 57.5)	75.2(66 to 82.5)	40.5(27.5 to 53.5, <0.001)	51.2(49 to 53.3)	45.4(43 to 47.8)	69.5(64.8 to 73.8)	44.2(37.2 to 51.2, <0.001)	3.7 (Y)
ARI	60.5(57.2 to 63.7)	59.6(56.1 to 62.9)	71.1(61.5 to 79.1)	20.1(7.3 to 33, 0.002)	45.4(43.2 to 47.5)	45.2(42.9 to 47.5)	47.7(41.8 to 53.5)	Non-linear¶	N/A
ORS	39(34.3 to 44.0)	40(34.9 to 45.2)	37.5(29.2 to 46.6)	Non-linear¶	21.7(20.1 to 23.4)	20.7(19.1 to 22.4)	25.1(21 to 29.6)	9.4(3.2 to 15.6, 0.003)	N/A

Intervention	2010/2011				2022/2023				2022/2023 vs 2010/2011
	Total coverage% (95% CI)	Rural%(95% CI)*	Urban% (95% CI)†	Urban minus rural (pp)**(95% CI, P value)	Total coverage% (95% CI)	Rural%(95% CI)*	Urban%(95% CI)†	Urban minus rural (pp)**(95% CI, P value)	2022/2023–2010/2011 (pp)CI overlap (Y/N)††
ANC4	14.6(13 to 16.3)	10.5(9 to 12.2)	32.8(28.9 to 37)	22.3(17 to 26.7, <0.001)	33.4(31.9 to 35)	30.1(28.4 to 31.8)	44.5(41.2 to 47.8)	14.4(10.7 to 18.1, <0.001)	−7.99 (Y)
SBA	38.7(35.8 to 41.6)	30.5(27.4 to 33.8)	74.4(70.4 to 78)	43.8(38.9 to 48.8, <0.001)	67.5(65.8 to 69.3)	61.1(59 to 63.2)	88.8(86.1 to 91.1)	27.7(24.4 to 31, <0.001)	−16.15 (N)
BCG	64.2(60.4 to 67.7)	61(56.6 to 65.2)	79.2(75.3 to 82.6)	18.2(12.5 to 23.8, <0.001)	64.7(62.4 to 67)	58.9(56.2 to 61.6)	83.2(79.4 to 86.4)	24.3(19.9 to 28.7, <0.001)	6.12 (Y)
DPT3	40.2(36.8 to 43.7)	37.5(33.4 to 41.6)	53.2(48.5 to 57.3)	15.5(9.5 to 21.6, <0.001)	51.3(48.9 to 53.8)	47.1(44.3 to 49.9)	64.9(60.2 to 69.4)	17.8(12.4 to 23.2, <0.001)	2.31 (Y)
MSL	55.5(51.7 to 59.1)	52.4(47.9 to 56.7)	70(65.4 to 74.2)	17.6(11.4 to 23.9, <0.001)	51.2(49 to 53.3)	47.5(45 to 49.9)	63(59.2 to 66.6)	15.5(12 to 19.9, <0.001)	−2.13 (Y)
ARI	60.5(57.2 to 63.7)	59.2(55.3 to 62.9)	67.3(63 to 71.4)	8.1(2.6 to 13.8, 0.005)	45.4(43.2 to 47.5)	45.5(43.3 to 47.7)	44.5(38.8 to 50.5)	−1(−7.3 to 5.3, 0.765)	−9.09 (Y)
ORS	39(34.3 to 44)	40.2(34.7 to 45.8)	32.7(28.8 to 36.8)	−7.4(−14.3 to −0.6, 0.033)	21.7(20.1 to 23.4)	20.1(18.5 to 21.7)	28.6(23.9 to 33.9)	8.6(3.3 to 13.8, 0.001)	16.01 (N)

Further stratification by wealth quintiles, maternal education level and area of residence. Comparison of the absolute inequalities.

* Coverage among the lowest strata, that is, poorest wealth quintile (Q1), none or pre primary education (EDU1) or rural residence.

† Coverage among the highest strata, that is, richest wealth quintile (Q5), secondary+ education (EDU3) or urban residence.

‡ Estimate of the level of absolute inequality via logistical regression, that is, Slope Index of Inequality (SII) in percentage points (pp).

§ Comparison of the SII 95% CIs from both surveys for the corresponding intervention and strata, Yes (Y) if overlapping, No (N) if no overlap.

¶ No linear relationship between the coverage level and the strata (wealth or education level) and thus not suitable for a logistic regression.

** Crude mathematical difference of urban minus rural coverage, 95% CIs via linear regression.

†† Comparison of the linear regression 95% CIs for the corresponding intervention and strata, Yes (Y) if overlapping, No (N) if no overlap.

ANC4, 4 or more antenatal care (ANC) visits; ARI, care seeking with a skilled provider for children with acute respiratory illness, that is, suspected pneumonia; BCG, Bacillus-Calmette-Guérin vaccination.; DPT3, 3 doses of at least a tetravalent diphtheria-pertussis-tetanus vaccination.; MSL, first dose of the measles vaccination schedule ; ORS, treatment of diarrhoeal diseases with oral rehydration solution or salts.; SBA, skilled birth attendance.

The educational inequalities display a slightly different pattern. While the SII remains high for both maternal indicators (ANC4 37 pp, 95% CI 31.6 to 42.3, p<0.001; SBA 44.2 pp, 95% CI 37.8 to 50.6, p<0.001), the largest absolute inequality was found among the vaccine indicators, especially BCG immunisation (52.7 pp, 95% CI 45.1 to 60.3, p<0.001) and DPT3 (52.7 pp, 95% CI 45.5 to 59.9, p<0.001). This contrasts the 2010/2011 study, where the intervention with the largest absolute inequality (SII) was SBA with 66.2 pp (95% CI 59.6 to 72.8, p<0.001) difference between most and least educated.

All indicators except for ARI presented a pro urban pattern. Again, the absolute inequalities were largest for SBA (27.7 pp, 95% CI 24.43 to 30.95, p<0.001) and smallest for the care-seeking indicator ORS (8.6 pp, 95% CI 3.34 to 13.8, p=0.001).

#### Socioeconomic inequalities in composite coverage

The SII of the CCI shows a significant pro rich and pro educated pattern for both studies where the economic inequalities increased by 5.4 pp (95% CI 4.9 to 5.6, p<0.001) and the educational inequalities slightly decreased over time (−1.1 pp, 95% CI −1.8 to −0.4, p=0.003) (table 3).

**Table 3:** CCI with corresponding 95% CI from jackknifing for each year and all three equity dimensions

	CCI 2010/2011% (95% CI)	SII 2010/2011 (pp)(95% CI, P value)	CCI 2022/2023% (95% CI)	SII 2022/2023 (pp)(95% CI, P value)	Difference in SII (pp)(95% CI, P value)
Total					
	42.1(40.3 to 44)		46.2(44.6 to 47.9)		
Wealth quintile					
Poorest	30.9(27.3 to 34.5)	28.1(27.7 to 28.6, <0.001)	31.8(29.2 to 34.4)	33.6(33.5 to 33.8, <0.001)	5.4(4.9 to 5.6, <0.001)
Second	37.9(35.1 to 40.7)		40.7(38.6 to 42.9)		
Third	39.8(37.1 to 42.5)		47.2(45.3 to 49.1)		
Fourth	46.2(43.8 to 48.5)		51.8(49.4 to 54.1)		
Richest	56.4(54 to 58.7)		62.7(60.2 to 65.1)		
Education level					
None or pre primary	40.3(38.4 to 42.1)	34.2(33.4 to 34.9, <0.001)	42.2(40.4 to 43.9)	33.1(33 to 33.2, <0.001)	−1.1(−1.8 to −0.4, 0.003)
Primary	52.1(48.1 to 56)		56.8(54.2 to 59.3)		
Secondary or higher	63.6(59.4 to 67.8)		61.4(59.2 to 63.6)		
Area of residence					
Urban	55.8(53.4 to 58.1)	N/A(Crude: 16.7%)*	57.4(54.8 to 60)	N/A(Crude: 14.5%)*	N/A(Crude: −2.2%)*
Rural	39.1(37 to 41.1)		42.9(41 to 44.7)		

Slope Index of Inequality (SII) for wealth and educational equity dimensions and corresponding differences.

* SII calculation is not applicable for urban/rural dimension. Displaying the crude difference of urban minus rural coverage and the difference in coverage from 2022/2023 minus the difference in coverage from 2010/2011, respectively.

CCI, Composite Coverage Index.

### Spatiotemporal analysis

#### Temporal changes in inequalities

Both maternal indicators (ANC4 and SBA) saw a significant increase in coverage, while the coverage of both care-seeking indicators significantly decreased.

As displayed in table 2, the only statistically significant change in the pattern of absolute wealth disparities is an 18.8 pp increase for BCG immunisation. Neither of the other six indicators showed a significant change over time.

**Figure 1:**
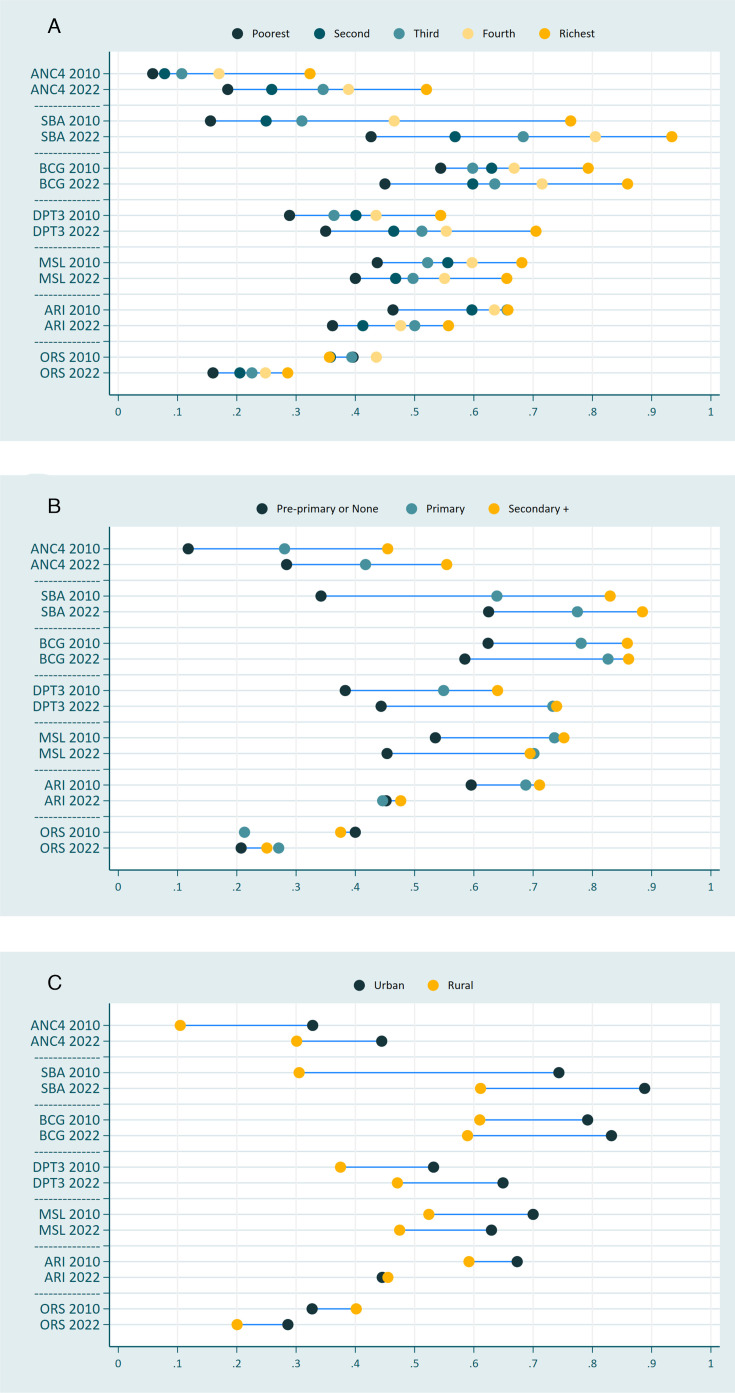
Equiplots for all three equity dimensions comparing both time points for each of the seven interventions. A connecting line indicates the presence of all individual substrata and is connecting lowest to highest values. (A) Equiplot comparing each intervention by the two time points stratified by wealth quintiles. (B) Equiplot comparing each intervention by the two time points stratified by maternal education level. (C) Equiplot comparing each intervention by the two time points stratified by area of residence. ANC4, 4 or more antenatal care (ANC) visits; DPT3, 3 doses of at least a tetravalent diphtheria-pertussis-tetanus vaccination; ORS, treatment of diarrhoeal diseases with oral rehydration solution or salts; SBA, skilled birth attendance; BCG, Bacillus-Calmette-Guérin vaccination; MSL, first dose of the measles vaccination schedule; ARI, care seeking with a skilled provider for children with acute respiratory illness, that is, suspected pneumonia.

Considering the maternal education status, only SBA showed a significant change, that is, a 22 pp decrease in SII. The level of educational inequality persisted for all three vaccinations and ANC4 (figure 1).

Coming from a pro rural configuration in 2010/2011, the coverage with ORS flipped to an urban-favouring pattern in 2022/2023 through a 16 pp increase in absolute inequality. SBA, on the other hand, while displaying the highest estimate of absolute inequalities, also showed the only significant decrease in absolute inequalities between the two studies in the urban/rural group (−16 pp).

A more nuanced depiction of the spatiotemporal differences can be found in the online supplemental appendix where we display equiplots for each of the seven indicators stratified by the three equity dimensions following the regional grouping (online supplemental figure 1) from the 2010/2011 MICS (online supplemental figure 2).

#### Regional coverage and inequalities

**Figure 2:**
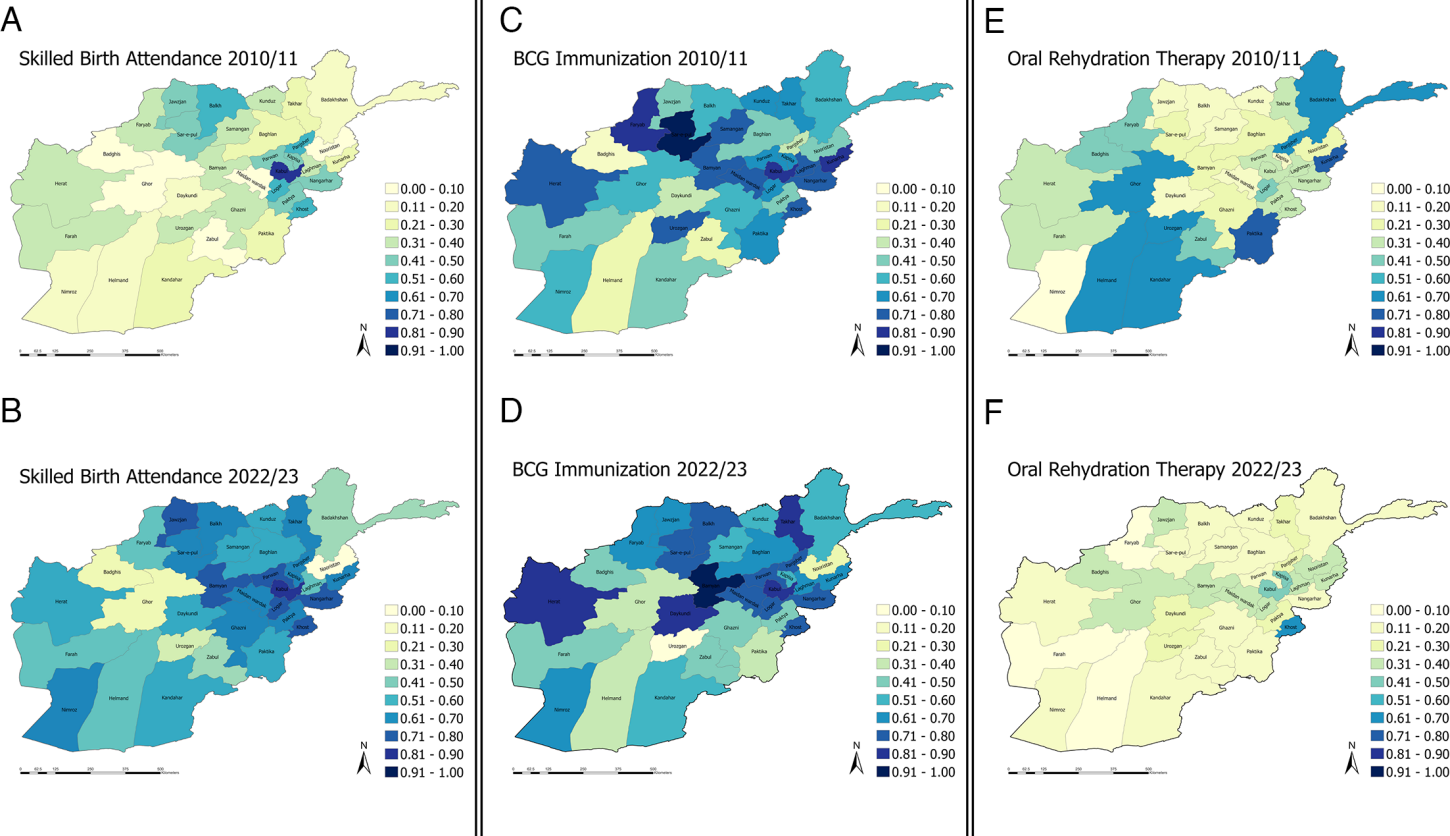
Spatial distribution of mean coverage rates for skilled birth attendance, BCG immunisation and oral rehydration therapy for each of the 34 Afghan provinces. Exact values with corresponding 95% CIs are included in online supplemental table 3. Skilled birth attendance: (A) mean coverage of skilled birth attendance per province in the 2010/2011 survey; (B) mean coverage of skilled birth attendance per province in the 2022/2023 survey. BCG immunisation: (C) mean coverage of BCG immunisation per province in the 2010/2011 survey; (D) mean coverage of BCG immunisation per province in the 2022/2023 survey. Oral rehydration therapy: (E) mean coverage of oral rehydration therapy per province in the 2010/2011 survey; (F) mean coverage of oral rehydration therapy per province in the 2022/2023 survey.

Coverage showed a high spatiotemporal variability. Figure 2A–F shows the mean provincial coverage for three proxy indicators (SBA, BCG and ORS) by year. SBA increased throughout most provinces, with Kabul exhibiting the highest coverage in both 2010 (81.4%, 95% CI 75.5% to 86.2%) and 2022 (94.2%, 95% CI 90.2% to 96.7%). Nooristan consistently had very low SBA coverage, showing minimal improvement. BCG immunisation rates mirrored the pattern, with Kabul the highest and Nooristan the lowest. 10 of 34 provinces experienced ≥10 pp reductions among BCG vaccination rates, with coverage in Urozgan dropping by 61 pp. In contrast, Daykundi more than doubled its BCG coverage rates in 2022/2023 (84.6%, 95% CI 76.3% to 90.4%).

ANC4 coverage was highest in Bamyan (63.2%, 95% CI 51.9% to 73.2%), while Urozgan showed the steepest decline (−22 pp). Measles and DPT3 coverage followed similar trends: Daykundi and Nimroz experienced the largest increases in both immunisation coverages (DPT3 +62.6 and 61.6 pp; MSL +45.9 and +59.2 pp, respectively). In 2022/2023, Bamyan had the highest overall coverages for both DPT3 (92.6%, 95% CI 80.7% to 97.4%) and MSL (88.7%, 95% CI 73.6% to 95.7%), while Urozgan recorded the lowest coverages for both vaccines (DPT3 3.4%, 95% CI 1.3% to 8.3%; MSL 8.6%, 95% CI 3.8% to 18.1%). MSL coverage declined in almost 60% of all provinces.

ARI coverage decreased in about half of the provinces, with Farah reporting the lowest coverage at 14.3% (95% CI 9.4% to 21.2%) (online supplemental figure 3 and table 3).

Due to intraprovincial heterogeneity across the seven indicators, identifying a single ‘best’ or ‘worst’ state of MCH coverage is not possible. However, online supplemental table 3 outlines following overall patterns.

In 2022/2023, Bamyan ranked among the highest coverage in both maternal indicators (ANC4 63.2%, 95% CI 51.9% to 73.2%; SBA 82.9%, 95% CI 75.1% to 88.6%), while Nooristan (ANC4 6.7%, 95% CI 3.9% to 11.3%; SBA 9.5%, 95% CI 6.8% to 13.2%) and Ghor (ANC4 8.6%, 95% CI 5.0% to 14.2%; SBA 21.0%, 95% CI 14.5% to 29.4%) ranked lowest. Nimroz recorded some of the most substantial improvements (ANC4 +34.4 pp, SBA +63.9 pp).

For vaccination coverage, Bamyan again ranked among the highest covered provinces in 2022/2023 (BCG 98.6%, 95% CI 94.7% to 99.6%; DPT3 92.6%, 95% CI 80.7% to 97.4%; MSL 88.7%, 95% CI 73.6% to 95.7%). Daykundi recorded one of the greatest increases (BCG +51.0 pp, DPT3 +62.6 pp, MSL +45.9 pp), alongside Nimroz (DPT3 +61.6 pp, MSL +59.2 pp). Urozgan had some of the lowest vaccination coverages in 2022/2023 (BCG 9.6%, 95% CI 4.7% to 18.5%; DPT3 3.4%, 95% CI 1.3% to 8.3%; MSL 8.6%, 95% CI 3.8% to 18.1%), while Paktika experienced the most severe declines (BCG −37.7 pp, DPT3 −24.3 pp, MSL −51.0 pp).

In treatment seeking, Helmand ranked among the lowest covered provinces in 2022/2023 (ARI 19.0%, 95% CI 13.1% to 26.6%; ORS 3.9%, 95% CI 2.6% to 5.7%), also recording the greatest declines between the two survey periods (ARI −72.4 pp, ORS −58.9 pp).

Overall, Bamyan, Nimroz and Daykundi stood out for their strong or improving coverage across multiple indicator groups, while Nooristan, Urozgan and Paktika repeatedly ranked among the lowest covered provinces or experienced the most severe declines.

#### Spatiotemporal trends in composite coverage

The national CCI in 2022/2023 was 46.2% (95% CI 44.6% to 47.9%), showing a statistically significant increase from the 2010/2011 CCI of 42.1% (95% CI 40.3% to 44.0%). The 2022/2023 CCI indicates that 46.2%, that is, less than half of the Afghan women and children, have access to these essential interventions. When only considering children born on or after 1 May 2021, the start of the Taliban’s offensive and withdrawal of international forces, the CCI for 2022/2023 decreases to 45.4% (95% CI 43.8% to 47.09%), showing overlapping CIs with the 2010/2011 CCI.

**Figure 3:**
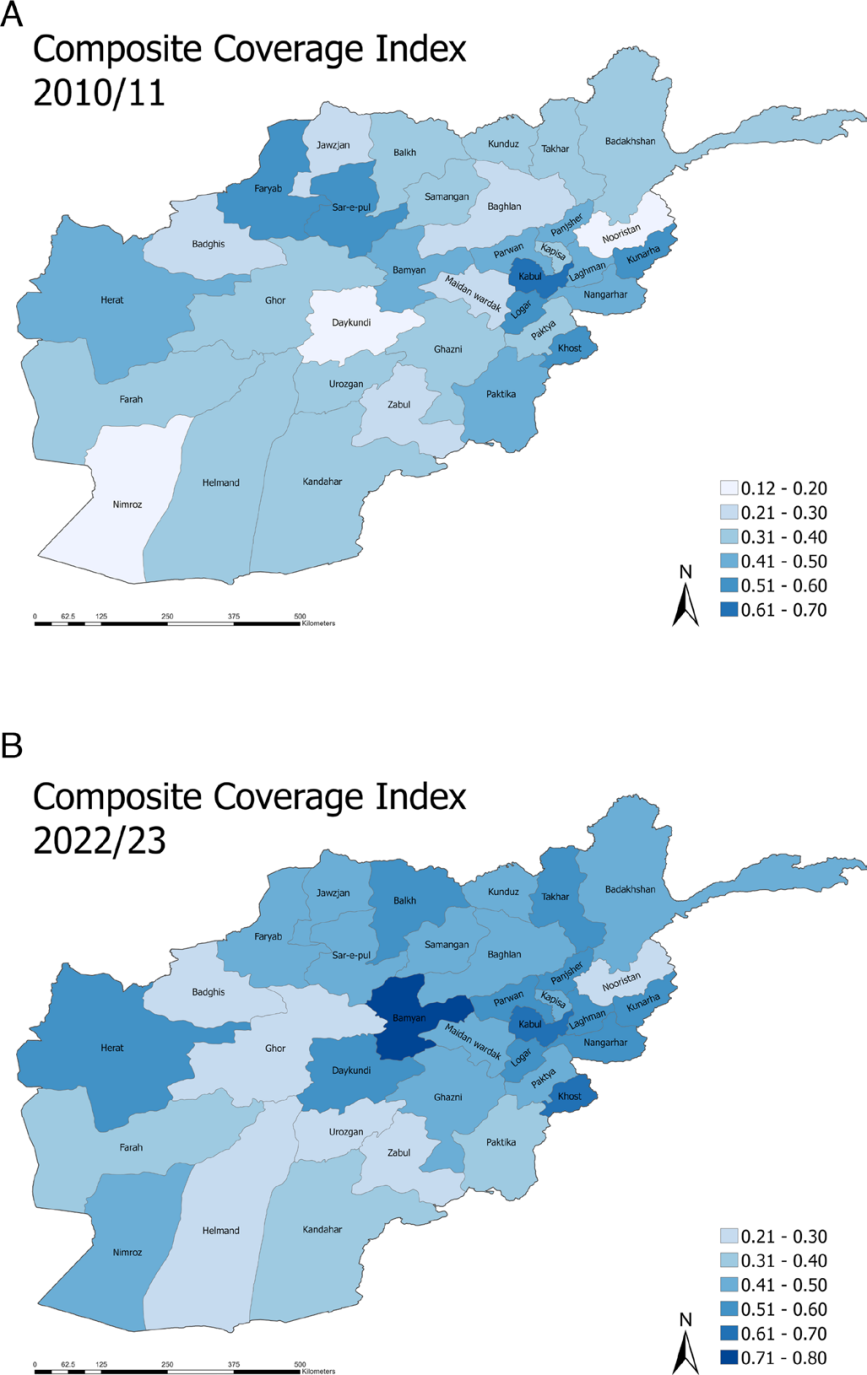
Spatial distribution of mean coverage rates for Composite Coverage Index (CCI) for each of the 34 Afghan provinces. Exact values are included in online supplemental table 4. (A) Mean CCI for each Afghan province in the 2010/2011 survey. (B) Mean CCI for each Afghan province in the 2022/2023 survey.

Geographically, the CCI and its change between the two studies varies widely (figure 3A,B). In 2022, the coverage was highest in Kabul (63.8%) and Bamyan (70.4%). The lowest indices could be found in Nooristan (21.5%), which has seen less than 4 pp improvement over the 12 years between the two surveys, and Urozgan (24.6%). Nimroz and Daykundi experienced an increase of 34.2 and 39.3 pp in CCI coverage, respectively. The largest decrease of around 10 pp was documented in Urozgan and Paktika (online supplemental table 4).

### Intersection of socioeconomic and spatiotemporal trends in inequalities

To visualise the extent and development of inequality over space and time, we used equiplots that showed a stark variation depending on the intervention (online supplemental figure 2).

While the overall CCI slightly improved, the variability is much higher on the regional level (online supplemental figure 4). Economic disparities in the Central, North and West regions did not show any notable improvement or change in patterns. On the contrary, the Central Highlands, East, North East and South East regions generally improved but maintained their level of inequality. Among the lowest education levels (online supplemental figure 4B), the CCI decreased in the West and South and showed noteworthy improvement in the East, North East and South East. A similar pattern applies when comparing urban and rural residences.

While the coverage in all SBA substrata improved between the surveys, the change in equality is mostly marginal, with the central region being the exception. SBA coverage improved especially for the least educated across all provinces. The improvement is less pronounced for higher education. The ubiquitous urban-favouring pattern in SBA improved across all regions. When comparing the inter-regional differences in 2022, the West persists in showing particularly low SBA coverage combined with a considerably large spread between strata groups.

Among the BCG immunisation coverage inequalities generally increased, and further, the most disadvantaged did not experience much or any improvement in overall coverage levels. Except for the Central Highlands, the disparities among wealth strata widened. Disparities between urban and rural residents either increased or remained at the same level.

Compared with the two indicators above, the coverage with oral rehydration therapy generally shows fewer inequalities. The coverage has dropped among most wealth strata except for the Central Highlands. Similarly, all regions besides the Central and the Central Highlands lost coverage among their education strata.

In summary of all seven interventions, the three eastern regions (online supplemental figure 1) stand out with repetitive loss in coverage across multiple interventions and strata, while the composite coverage dropped notably in the South.

## Discussion

To our knowledge, this spatiotemporal analysis is the first study of its kind after the political takeover by the Taliban in August 2021. Despite some improvements in overall coverage, such as the increase in the national CCI by 4 pp over 12 years, the situation in Afghanistan’s healthcare remains critical. When including only children born after 1 May 2021—the beginning of the Taliban’s offensive—the CCI difference between 2010/2011 and 2022/2023 is only 3.3 pp and is not statistically significant. While omitting children born before 1 May from the CCI calculations limits the comparability of the indices due to the different retrospective intervals of some indicators, the comparison allows a glimpse into the new status quo. It suggests that the apparent slight improvement may actually reflect stagnation or decay rather than true progress.

Previous analyses used the 2018 Afghan Health Survey (AHS) to compute the CCI.^38^ Based on the AHS values and using the adapted CCI formula, we calculated a national CCI of 53.14% from the AHS 2018 data.^39^ While this calculation is only meant to serve as a midpoint reference between our two data points, this would indicate that major progress was lost between 2018 and 2022/2023.

However, while the current status quo is far from satisfactory, it is worth noting that given Afghanistan’s turbulent history, the situation could be much worse. Especially surprising is the large overall increase in coverage of maternal interventions ANC4 and SBA and the decrease in educational inequalities for SBA. This progress is counterbalanced by a significant decline in coverage of care-seeking interventions, specifically for ARI and ORS treatment.

The persistence of socioeconomic inequalities, particularly in wealth and education, highlights a systemic issue and is consistent with past studies.^2 38 40 41^ Economic disparities were most pronounced in SBA coverage and even increased for BCG immunisation as well as for the national CCI. Educational disparities were largest in vaccination coverage. Notably, the educational inequality in SBA and CCI coverages decreased, an exception that requires further investigation to understand its underlying causes.

Compared with other LMIC studies, the persistent economic inequalities are not surprising,^42^ but the developments over time reported in this study largely contrast with global and regional trends recently analysed across 101 countries between 1993 and 2021. That analysis indicated a steady global increase in CCI alongside a decline in economic inequalities, with the most pronounced improvements in South Asia. In stark contrast, our findings suggest that Afghanistan is deviating from these trends, both at the regional and global levels.^43^

Given many reports on gender-based marginalisation,^6 7 20 44^ on the other hand, one could hypothesise that female education is systematically neglected to a point where it can no longer serve as a reliable predictor of healthcare access. In 2019, Afghanistan already ranked 157 out of 162 in the Gender Inequality Index, and the Taliban’s rule may reverse part of the little progress that has been made in the past decades.^44^ All indicators analysed in this study rely fundamentally on maternal participation and healthcare-seeking behaviour—from ANC access to managing childhood illnesses—highlighting women’s pivotal role in health system functionality. Systemic gender disparities in healthcare access and decision-making autonomy therefore pose critical risks, as failure to address these issues may significantly exacerbate existing challenges.

The high interprovincial variation in vaccination coverage requires further investigation. Security and accessibility challenges have limited the spatial resolution of previous studies, hindering a comprehensive explanation of the disparities observed in our analysis.^45^ However, a 2017 study offers some insights: Bamyan, with high coverage in our study, already had a low zero immunisation rate, facilitating further improvements. Similarly, improvements in Daykundi and Nimroz may relate to higher maternal education levels and low zero immunisation rates. In contrast, Urozgan’s low rates may stem from low maternal education, a high dropout rate (38%) and long travel distances to vaccination sites. The sharp decline in immunisation coverage in Paktika could be attributed to low maternal education and sociocultural barriers such as restrictions on mothers vaccinating children without a mahram (male companion).^46^

Paktika’s situation is particularly concerning considering that a measles outbreak occurred shortly before data collection, and despite targeted WHO efforts, coverage remains over 50 pp lower than in 2010/2011.^47^

The disparities described above, coupled with stagnation in progress, underscore the urgent need for intervention. The drastic reduction in international funding, particularly coinciding with the Taliban’s return to power, is associated with significant challenges in Afghanistan’s healthcare system, especially for its most vulnerable populations—women and children.

Therefore, policy implications are clear: the World Bank, as administrator of the ARTF, and its primary donors—including the European Union, Japan and the USA—must prioritise restoring and expanding funding streams for the Basic Package of Health Services and Essential Package of Health Services with time-bound commitments and a focus on the distinct needs of diverse population groups.^16 23^ Targeted interventions should prioritise underserved rural provinces like Nooristan and Urozgan, as well as urban populations living in informal settlements or facing socioeconomic hardship. Socioeconomic stratification must also guide resource allocation, ensuring that the poorest and least educated populations receive adequate support.

Although the World Bank has resumed partial funding after 2 years of suspension, current support remains low compared with pre-2021 levels,^48 49^ and the global trend of shrinking development budgets among major donors poses a significant challenge to fully restoring previous funding levels. Given these fiscal constraints, recommendations are primarily directed at international donors and multilateral agencies, as domestic revenue generation in Afghanistan remains extremely limited due to the ongoing economic crisis and lack of recognised government structures.^50^ In the absence of substantial domestic funding, the health system—and especially services for women and children—will remain heavily dependent on external support. Without renewed and sustained international investment, critical gaps in healthcare provision for Afghanistan’s most vulnerable populations are likely to widen further.

Given the increasing barriers preventing women and mothers from accessing healthcare in Afghanistan, especially in rural areas, international organisations and NGOs must focus on practical strategies to reduce these obstacles. While systemic change may be limited under current policies, NGOs can help mitigate the impact by bringing healthcare services closer to communities.

Mobile health units, for example in Haiti, have shown to be as efficient as fixed clinics while delivering care to remote populations.^51 52^ Community-based and community outreach programmes in Bangladesh and Pakistan have shown to reduce home deliveries and enhance ANC.^53 54^ Community transport schemes can further bridge distances, as shown in Bangladesh’s Rohingya camps.^55^

These NGO-led mobile, outreach and transport interventions bring maternal services to isolated women, helping to bridge coverage gaps that the fragile state system cannot fill.

In addition, the international humanitarian and development sectors must collaborate to mitigate the impact of restrictive policies on healthcare by addressing gaps left by the declining workforce. Tailored immunisation campaigns should address coverage gaps in rural areas while ensuring equitable access in urban areas. International organisations, NGOs and civil society must advocate for women’s rights and improving healthcare access across all socioeconomic groups. With healthcare as one of the last remaining employment options for women, especially in key areas like obstetrics and gynaecology,^56^ healthcare NGOs have a small but crucial opportunity to promote female engagement. These actions are profoundly linked to the overall health outcomes of the Afghan population as the systemic neglect of women’s health and education is likely worsening existing inequalities and poses a significant threat to public health, particularly maternal and child mortality.

### Strengths and limitations

To our knowledge, this spatiotemporal comparison is the first study of its kind after the political takeover of the Taliban in August 2021 and the first to analyse the new MICS 2022/2023. Hence, it contributes new evidence to the existing literature, which almost exclusively relies on pre-Taliban data and is subsequently outdated. Our comparative approach is another major strength of this study as it allowed us to link two large-scale national health surveys and to analyse the change of the Afghan health system over more than a decade. We tailored our analysis by employing multiple approaches to assess changes across strata, time and space. This flexibility enabled us to adapt our methodology to varying data availability and sample sizes, ensuring the highest level of precision and robustness in our investigation of each indicator and index.

However, our study poses several limitations. Due to the data points being set between 2010 and 2022, this study cannot determine whether significant progress between 2010 and August 2021 was reversed during the transition period and the following regime change, or if the country’s healthcare development remained stagnant during the entire time frame. To address this, we incorporated findings from the 2018 AHS.^39^

Due to the missing family planning data in the 2022/2023 questionnaire, we had to drop the reproductive health data that are traditionally included in the CCI, which made the comparison with other publications impractical. It is due to the modification of the CCI that we decided to entirely reanalyse the MICS 2010/2011 to ensure unbiased comparability. However, based on the neglect of female independence and health,^6^ we hypothesise that the CCIs presented in this paper may be overestimating the values of a complete, reproductive health inclusive, CCI. Furthermore, comparing two cross-sectional samples limited the statistical comparability of the CCI, as it comprised different individuals both years. We assessed this issue by jackknifing and comparing the CIs. Lastly, this study does not control for potential confounders like conflict intensity, distance to healthcare infrastructure and the location of displaced populations. Based on the reports we discussed in the introduction, these contextual settings may vary widely between the two study periods.

Future research is needed to determine the full impact the regime change has had on MCH and especially reproductive health. To allow for a thorough analysis of change over time on the personal level, a true longitudinal cohort study would be needed. Such a study could also account for confounding factors on the personal level and assess the impact of geographically confined health interventions. Additional research is also needed on the influence of the potential confounders we describe above.

## Conclusion

In conclusion, despite some improvement, this study unveils an alarmingly low coverage of MCH services all over Afghanistan: fewer than half of Afghan women and children have access to life-saving services while the previously described patterns of inequality persist.^2 40^

Higher education is associated with higher MCH coverage, emphasising the importance of education programmes. Our findings also underline the pressing needs of the poorest mothers and children living in rural areas. While every intervention needs improvement, the largest inequalities were found for SBA and BCG immunisation which is applied directly after birth.

Whether the Afghan health system is stagnant or deteriorating, these findings carry profound implications for policy and programming. They show a potential association between reduced international funding, systematic marginalisation of women and poor health outcomes among mothers and children. Future programme planning should prioritise provinces with the lowest CCI like Nooristan and Urozgan for immediate intervention, tailoring programmes to address the specific deficits in MCH services within those regions. Additionally, efforts should focus on mitigating socioeconomic disparities by implementing targeted interventions, such as subsidised healthcare access, designed to reach the poorest families and reduce financial barriers to essential services. Civil society and (international) NGOs must advocate against the marginalisation of women, employing female staff whenever possible. If not for ethical considerations alone, the new regime must recognise the economic ramifications of this trend, including significant losses through out-of-pocket healthcare expenditures, disability-adjusted life years and other socioeconomic factors. Inaction is no longer an option; decisive action is imperative to safeguard the health and well-being of Afghanistan’s most vulnerable populations.

## Supplementary material

10.1136/bmjgh-2024-018577 online supplemental file 1

## Data Availability

Data are available in a public, open access repository.
