# Supplementary material for: Equity of maternal and child health services in Afghanistan: a spatiotemporal analysis of national survey datasets
Source: BMJ Glob Health. 2025 Jul 10;10(Suppl 3):e018577. doi: 10.1136/bmjgh-2024-018577 (PMC12258338; doi:10.1136/bmjgh-2024-018577)
Supplement: online supplemental file 1 [file bmjgh-10-Suppl_3-s001.pdf]

ONLINE SUPPLEMENTAL APPENDIX

SUPPLEMENTAL METHODS

STUDY SETTING

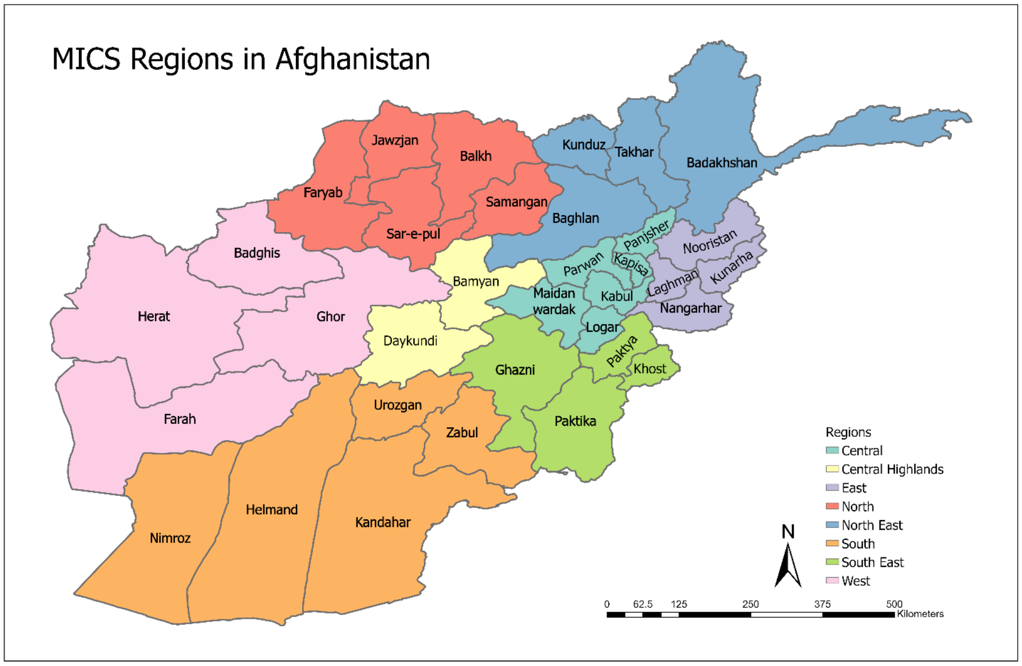

Supplemental Figure 1 - Composition of regions in Afghanistan according to the 2010/11 MICS report.[1]

INDICATOR SELECTION

Indicator	Denominator (number of)	Numerator (number of)
ANC4	Women of reproductive age with a livebirth in the past two years	Women reporting four or more antenatal care visits
SBA	Women of reproductive age with a livebirth in the past two years	Deliveries attended by a skilled provider (doctor, nurse, midwife, and/or auxiliary midwife)
BCG	Children between 12 and 23 months of age	Children receiving a single dose of a Bacillus Calmette-Guérin vaccination (according to card or caretaker's recall)
DPT3	Children between 12 and 23 months of age	Children receiving all three doses of an at least tetravalent diphtheria, pertussis, and tetanus vaccine (according to card or caretaker's recall)
MSL	Children between 12 and 23 months of age	Children receiving the first dose of the live measles vaccination (according to card or caretaker's recall)
ARI	Children $\leq$ 5 years of age with suspected pneumonia in the past 2 weeks (ill with cough plus tachypnea/dyspnea, whose symptoms were perceived to come from the chest $\pm$ blocked or runny nose)	Children receiving advice/treatment from a health facility or provider (defined as all public or private health facilities and providers except for private pharmacies)
ORS	Children $\leq$ 5 years of age with a diarrheal disease in the past 2 weeks	Children receiving oral rehydration solution or salt and continued feeding (defined as continued solid food or continued drinking if still breastfed)

Supplemental Table 1 - Indicator definitions in accordance with the Countdown definitions [2,3] and those used in the MICS reports from 2010/11[1] *and 2022/23*[4].

EQUITY ANALYSIS

EQUITY DIMENSIONS

To assess socioeconomic inequalities, we defined three socioeconomic equity dimensions: First, and especially in the context of women being denied access to higher education in the past two years, we assessed differences by maternal education level. Due to small sample sizes in higher education groups and in an effort to increase power, we summarized the five education groups presented in the 2022/23 data sets into three – no or pre-primary education (EDU1), primary education (EDU2), and secondary education or higher (EDU3). This condensation also ensured comparability to the 2010/11 data. Second, and as an economic indicator, we used wealth quintiles displaying the poorest (Q1) to the richest (Q5) quintile. Third, and as a measure of geographic classification and remoteness, we furthermore stratified by urban or rural living environment.

COMPOSITE COVERAGE INDEX CALCULATIONS

To calculate the CCI each indicator was separately calculated for the national level, each province, and each region and then further stratified. The sampling weights were included in each baseline calculation. We then used the already weighted proportions to calculate the CCI utilizing the adapted formula displayed. To ensure adequate power, we excluded proportions drawn from sample sizes smaller than 30 individuals. Subsequently, when one or more indicators were absent due to small sample sizes for further calculations of the CCI, the entire index was dropped from the analysis. However, to allow for a temporal comparison for each province, we did not apply the sample size restriction when calculating the CCI by province from the 2010/11 data. In contrast, the comparison of the CCI by region allowed for robust sample sizes by reducing spatial precision.

Because the CCI is an aggregated index that is not tied to the individual level, we calculated the confidence intervals for the CCI via a jackknifing approach at the cluster level ($n_{cluster} = 449$ in 2010/11, $n_{cluster} = 982$ in 2022/23). Small cluster sample sizes for less densely populated or more rural regions did not allow this approach for the province level, therefore this study only presents crude values at this level. The aggregated nature and different composition of the underlying population of the CCIs over time limited the statistical comparability of the indices. As a result, we found regression or t-testing approaches to be inappropriate and only provide the crude differences and comparison of confidence intervals in this paper. Similarly to the direct comparison of SII, we considered any non-overlapping confidence interval as statistically significant.

WEIGHTING

Sampling weights were originally calculated based on sampling probabilities for each sample's primary sampling unit (PSU), considering additional factors like the number of households in an enumeration area (EA). These weights were then adjusted for non-response and standardized. [1,4]

SUPPLEMENTAL RESULTS

WEALTH	2010/11					2022/23					2022/23 vs. 2010/11
**Intervention**	Total Coverage (%), (95% CI)	Q1 (%) (95% CI) [1]	Q5 (%) (95% CI) [2]	Q5–Q1 (%)[3]	SII (p.p.) (95% CI, p-value) [4]	Total Coverage (%), (95% CI)	Q1 (%) (95% CI) [1]	Q5 (%) (95% CI) [2]	Q5–Q1 (%)[3]	SII (p.p.) (95% CI, p-value) [4]	SII difference (p.p.) (CI overlap Y/N) [6]
ANC4	14.6 (13, 16.3)	5.8 (3.9, 8.6)	32.4 (28.9, 36)	26.6	29.6 (24.6, 34.1, p < 0.001)	33.4 (31.9, 35)	18.5 (16.2, 21)	52 (48.2, 55.8)	33.5	36.9 (32.1, 41.7, p < 0.001)	7.3 (Y)
SBA	38.7 (35.8, 41.6)	15.6 (12.4, 19.4)	76.3 (72.9, 79.4)	60.7	63.2 (57.8, 68.5, p < 0.001)	67.5 (65.8, 69.3)	42.7 (39.4, 46)	93.4 (91.4, 95)	50.8	56.9 (53, 60.8, p < 0.001)	-6.3 (Y)
BCG	64.2 (60.4, 67.7)	54.4 (46, 62.5)	79.3 (74.6, 83.3)	24.9	24.9 (13, 36.8, p < 0.001)	64.7 (62.4, 67)	45 (39.8, 50.4)	85.9 (82.8, 88.6)	40.9	43.6 (37.3, 50, p < 0.001)	18.8 **(N)**
DPT3	40.2 (36.8, 43.7)	28.9 (22.5, 36.2)	54.4 (49.4, 59.2)	25.5	25.2 (14.7, 35.6, p < 0.001)	51.3 (48.9, 53.8)	35 (30.3, 40)	70.5 (65.9, 74.7)	35.5	37.2 (29.9, 44.6, p < 0.001)	12.1 (Y)
MSL	55.5 (51.7,59.1)	43.7 (35.3, 52.5)	68.1 (63.1, 72.7)	24.4	24.7 (12.8, 36.6, p < 0.001)	51.2 (49, 53.3)	40 (35.6, 44.5)	65.6 (61, 69.8)	25.6	27.8 (20.7, 35, p < 0.001)	3.1 (Y)
ARI	60.5 (57.2, 63.7)	46.4 (39.5, 53.3)	65.7 (59.9, 71.2)	19.3	19.1 (9.5, 28.8, p < 0.001)	45.4 (43.2, 47.5)	36.2 (32.8, 39.7)	55.7 (48.8, 62.5)	19.6	20.5 (13.3, 27.8, p < 0.001)	1.4 (Y)
ORS	39 (34.3, 44)	39.6 (32.5, 47.2)	35.6 (30.9, 40.7)	-4	Non-linear[5]	21.7 (20.1, 23.4)	16 (13.7, 18.6)	28.6 (23.7, 34)	12.6	13.5 (7.6, 19.5, p < 0.001)	N/A
**EDUCATION**	**2010/11**					**2022/23**					**2022/23 vs. 2010/11**
**Intervention**	Total Coverage (%), (95% CI)	EDU1 (%) (95% CI) [1]	EDU3 (%) (95% CI) [2]	EDU3-EDU1(%)[3]	SII (p.p.) (95% CI, p-value) [4]	Total Coverage (%) (95% CI)	EDU1 (%) (95% CI) [1]	EDU3 (%) (95% CI) [2]	EDU3-EDU1(%)[3]	SII (p.p.) (95% CI, p-value) [4]	SII difference (p.p.) (CI overlap Y/N) [6]
ANC4	14.6 (13, 16.3)	11.8 (10.4, 13.3)	45.4 (39.1, 52)	22.3	39.5 (33.6, 45.4, p < 0.001)	33.4 (31.9, 35)	28.4 (26.9, 30)	55.4 (51.5, 59.3)	27	37 (31.6, 42.3, p < 0.001)	-2.5 (Y)
SBA	38.7 (35.8, 41.6)	34.2 (31.4, 37.2)	83 (77.7, 87.2)	48.8	66.2 (59.6, 72.8, p < 0.001)	67.5 (65.8, 69.3)	62.5 (60.5, 64.4)	88.5 (85.9, 90.6)	26	44.2 (37.8, 50.6, p < 0.001)	-22 **(N)**
BCG	64.2 (60.4, 67.7)	62.4 (58.4, 66.2)	85.9 (77.8, 91.4)	23.5	40.8 (27.7, 53.8, p < 0.001)	64.7 (62.4, 67)	58.5 (55.8, 61.1)	86.1 (82.2, 89.3)	27.6	52.7 (45.1, 60.3, p < 0.001)	12 (Y)
DPT3	40.2 (36.8, 43.7)	38.3 (34.6, 41.9)	64 54.9, 72.2)	25.7	38.3 (26.2, 50.5, p < 0.001)	51.3 (48.9, 53.8)	44.4 (41.7, 47.1)	74 (69, 78.3)	29.6	52.7 (45.5, 59.9, p < 0.001)	14.4 (Y)
MSL	55.5 (51.7,59.1)	53.5 (49.5, 57.5)	75.2 (66, 82.5)	21.7	40.5 (27.5, 53.5, p < 0.001)	51.2 (49, 53.3)	45.4 (43, 47.8)	69.5 (64.8, 73.8)	24.2	44.2 (37.2, 51.2, p < 0.001)	3.7 (Y)
ARI	60.5 (57.2, 63.7)	59.6 (56.1, 62.9)	71.1 (61.5, 79.1)	11.5	20.1 (7.3, 33, p = 0.002)	45.4 (43.2, 47.5)	45.2 (42.9, 47.5)	47.7 (41.8, 53.5)	2.5	Non-linear[5]	N/A
ORS	39 (34.3, 44.0)	40 (34.9, 45.2)	37.5 (29.2, 46.6)	-2.5	Non-linear[5]	21.7 (20.1, 23.4)	20.7 (19.1, 22.4)	25.1 (21, 29.6)	4.3	9.4 (3.2, 15.6, p = 0.003)	N/A

AREA	2010/11				2022/23				2022/23 vs. 2010/11
**Intervention**	Total Coverage (%), (95 CI)	Rural (%) (95% CI) [1]	Urban (%) (95% CI) [2]	Urban-Rural (p.p.)[7]	Total Coverage (%) (95% CI)	Rural (%) (95% CI) [1]	Urban (%) (95% CI) [2]	Urban-Rural (p.p.)[7]	2022/23 - 2010/11 (p.p.)(CI overlap Y/N) [8]
ANC4	14.6 (13, 16.3)	10.5 (9, 12.2)	32.8 (28.9, 37)	22.3 (17, 26.7, p < 0.001)	33.4 (31.9, 35)	30.1 (28.4, 31.8)	44.5 (41.2, 47.8)	14.4 (10.7, 18.1, p < 0.001)	-7.99 (Y)
SBA	38.7 (35.8, 41.6)	30.5 (27.4, 33.8)	74.4 (70.4, 78)	43.8 (38.9, 48.8, p < 0.001)	67.5 (65.8, 69.3)	61.1 (59, 63.2)	88.8 (86.1, 91.1)	27.7 (24.4, 31, p < 0.001)	-16.15 **(N)**
BCG	64.2 (60.4, 67.7)	61 (56.6, 65.2)	79.2 (75.3, 82.6)	18.2 (12.5, 23.8, p < 0.001)	64.7 (62.4, 67)	58.9 (56.2, 61.6)	83.2 (79.4, 86.4)	24.3 (19.9, 28.7, p < 0.001)	6.12 (Y)
DPT3	40.2 (36.8, 43.7)	37.5 (33.4, 41.6)	53.2 (48.5, 57.3)	15.5 (9.5, 21.6, p < 0.001)	51.3 (48.9, 53.8)	47.1 (44.3, 49.9)	64.9 (60.2, 69.4)	17.8 (12.4, 23.2, p < 0.001)	2.31 (Y)
MSL	55.5 (51.7,59.1)	52.4 (47.9, 56.7)	70 (65.4, 74.2)	17.6 (11.4, 23.9, p < 0.001)	51.2 (49, 53.3)	47.5 (45, 49.9)	63 (59.2, 66.6)	15.5 (12, 19.9, p < 0.001)	-2.13 (Y)
ARI	60.5 (57.2, 63.7)	59.2 (55.3, 62.9)	67.3 (63, 71.4)	8.1 (2.6, 13.8, p = 0.005)	45.4 (43.2, 47.5)	45.5 (43.3, 47.7)	44.5 (38.8, 50.5)	-1 (-7.3, 5.3, p = 0.765)	-9.09 (Y)
ORS	39 (34.3, 44)	40.2 (34.7, 45.8)	32.7 (28.8, 36.8)	-7.4 (-14.3, -0.6, p = 0.033)	21.7 (20.1, 23.4)	20.1 (18.5, 21.7)	28.6 (23.9, 33.9)	8.6 (3.3, 13.8, p = 0.001)	16.01 **(N)**

Supplemental Table 2 - Coverage as proportions of each individual indicator for both surveys. Further stratification by wealth quintiles, maternal education level, and area of residence. Comparison of the absolute inequalities.

1 – Coverage among the lowest strata, i.e., poorest wealth quintile (Q1), none or pre-primary education (EDU1), or rural residence.

2 – Coverage among the highest strata, i.e., richest wealth quintile (Q5), secondary+ education (EDU3), or urban residence.

3 – Crude, mathematical difference in the coverage, i.e., highest minus lowest strata

4 – Estimate of the level of absolute inequality via logistical regression, i.e., slope index of inequality (SII) in percentage points (p.p.)

5 – No linear relationship between the coverage level and the strata (wealth or education level) and thus not suitable for a logistic regression

6 – Comparison the SII's 95% confidence intervals from both surveys for the corresponding intervention and strata, Yes (Y) if overlapping, No (N) if no overlap

7 – Crude mathematical difference of urban minus rural coverage, 95% confidence intervals via linear regression

8 – Comparison of the linear regression's 95% confidence intervals for the corresponding intervention and strata, Yes (Y) if overlapping, No (N) if no overlap

Skilled Birth Attendance

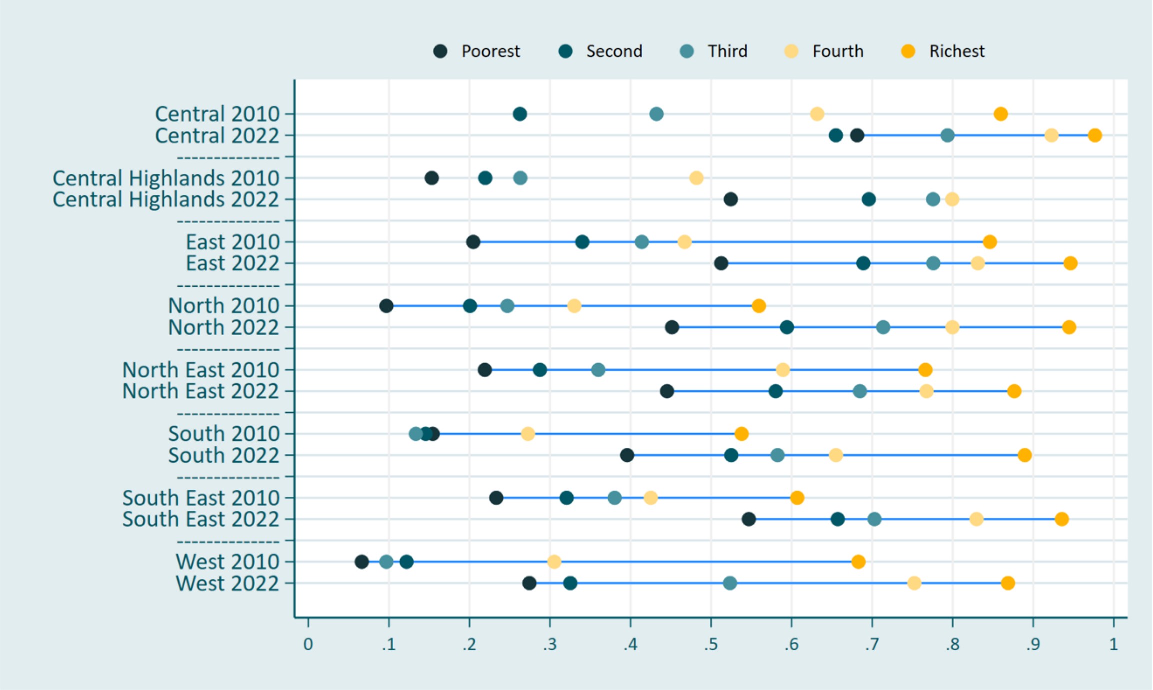

Supplemental Figure 2A - Equiplot of skilled birth attendance coverage within wealth quintiles and for each geographic region displayed in Figure 1.

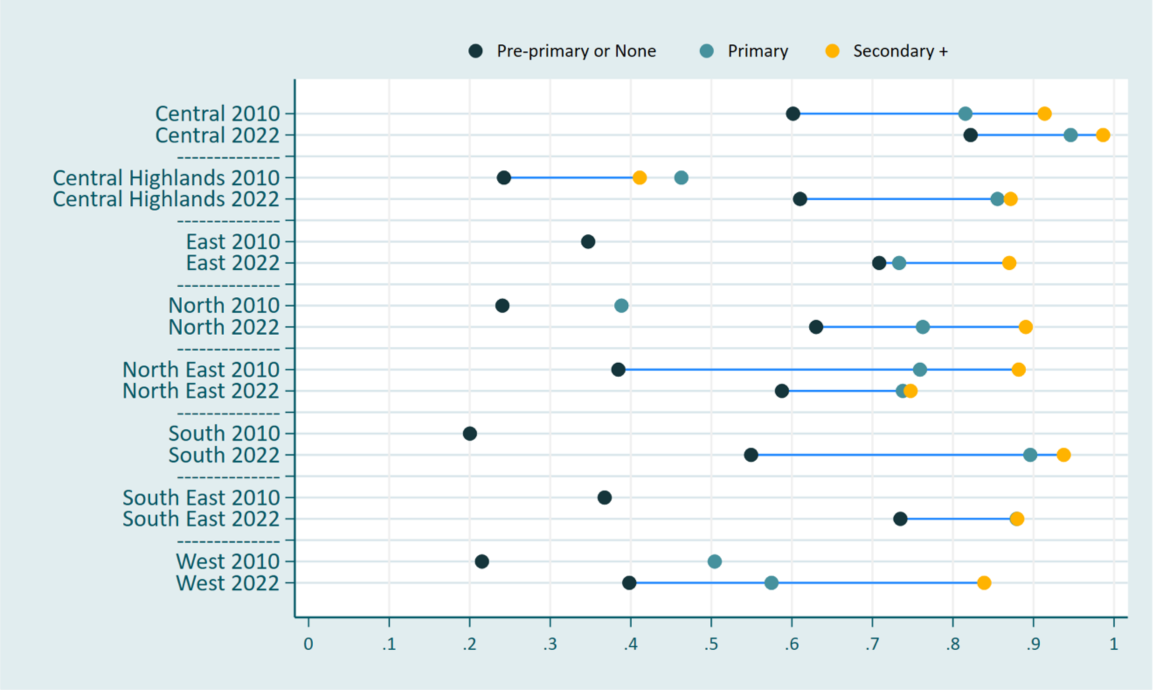

Supplemental Figure 2B - Equiplot of skilled birth attendance coverage within education groups and for each geographic region displayed in Supplemental Figure 1.

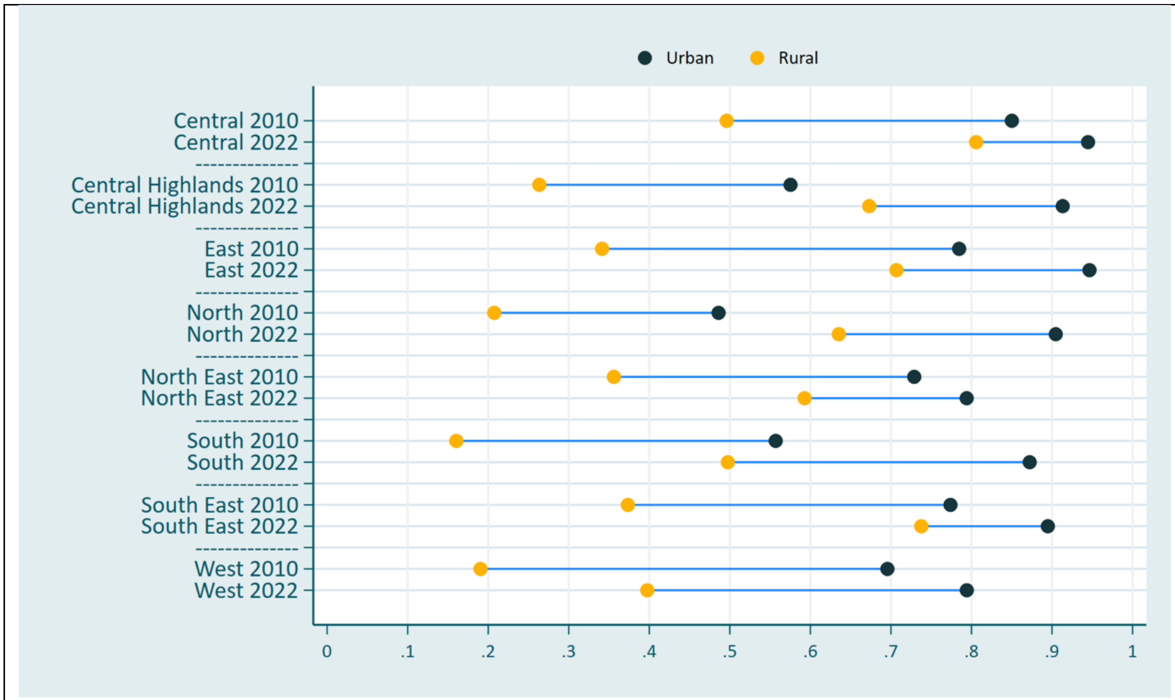

Supplemental Figure 2C- *Equiplot of skilled birth attendance coverage within urban or rural residence and for each geographic region displayed in Supplemental Figure 1.*

BCG Immunization

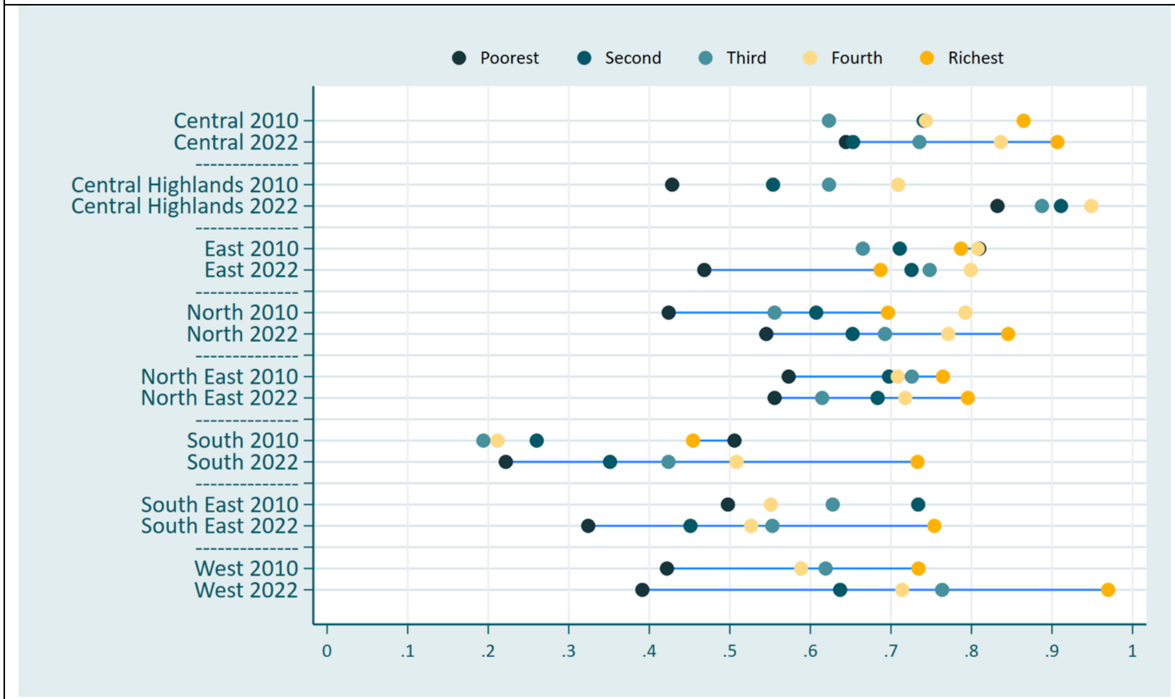

Supplemental Figure 2D - Equiplot of BCG immunization coverage within wealth quintiles and for each geographic region displayed in Supplemental Figure 1.

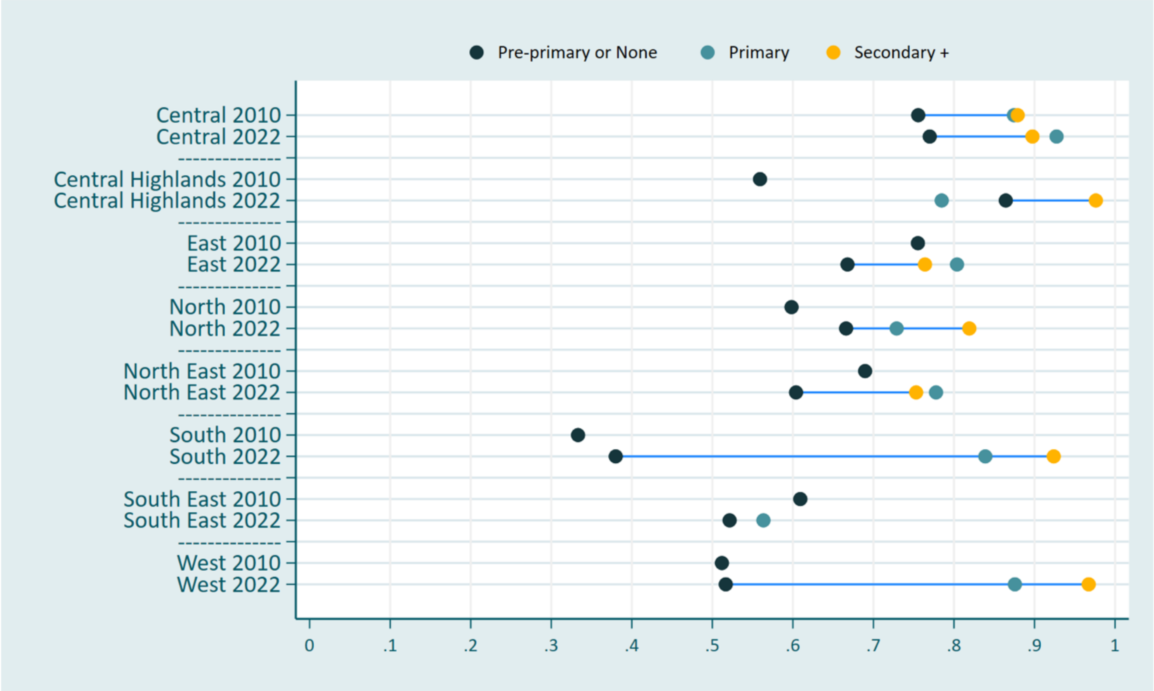

Supplemental Figure 2E - Equiplot of BCG immunization coverage within education groups and for each geographic region displayed in Supplemental Figure 1.

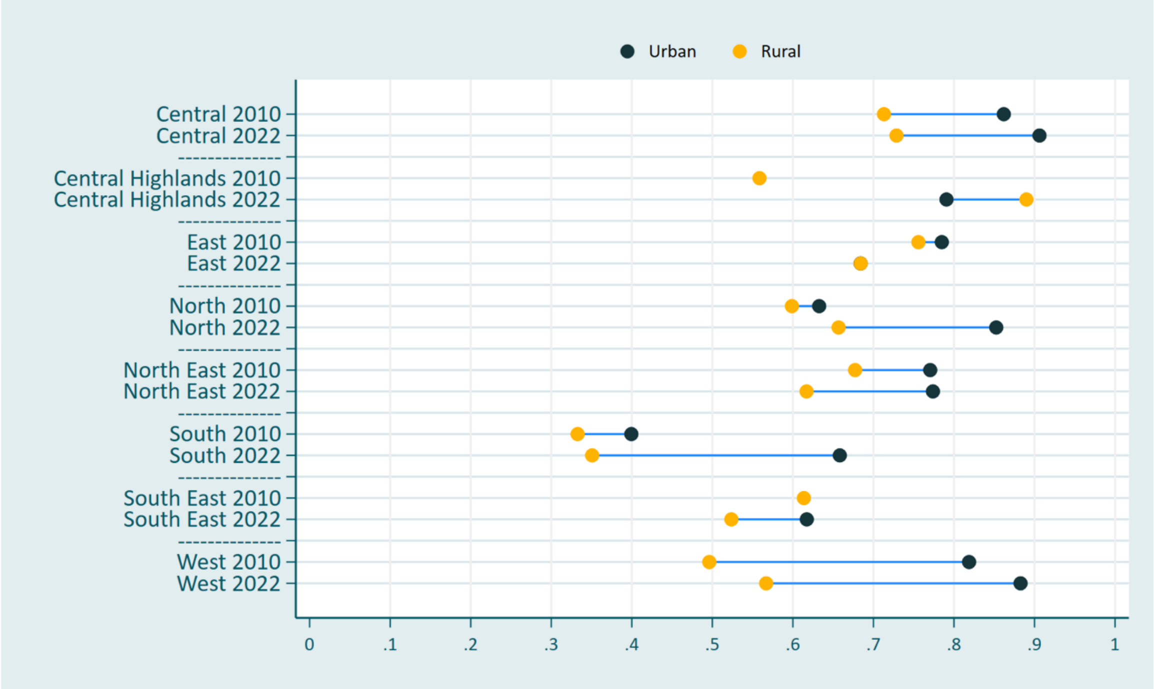

Supplemental Figure 2F - *Equiplot of BCG immunization coverage within urban or rural residence and for each geographic region displayed in Supplemental Figure 1.*

Oral Rehydration Therapy

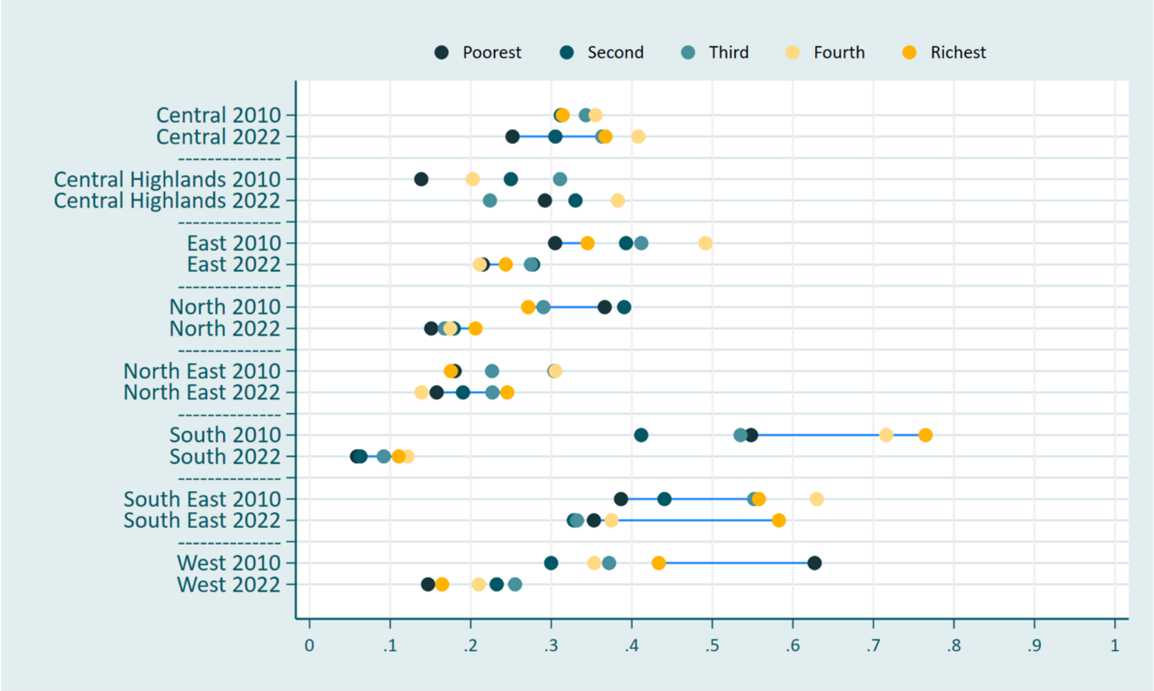

Supplemental Figure 2G - Equiplot of oral rehydration therapy coverage within wealth quintiles and for each geographic region displayed in Supplemental Figure 1.

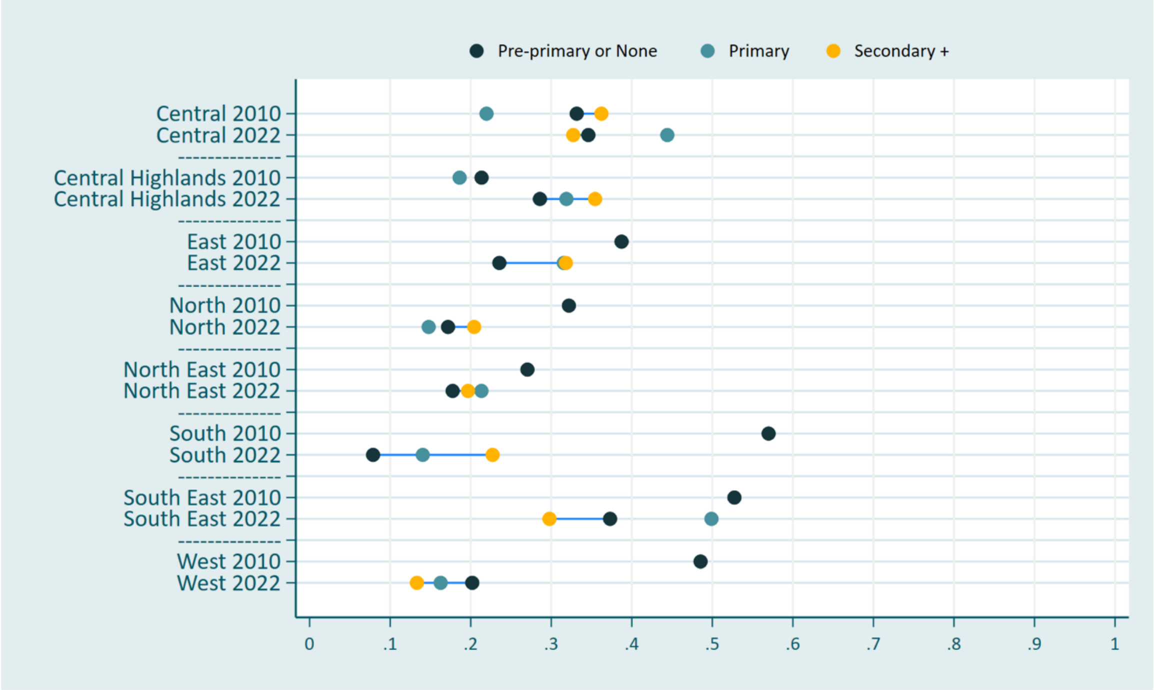

Supplemental Figure 2H - Equiplot of oral rehydration therapy coverage within education groups and for each geographic region displayed in Supplemental Figure 1.

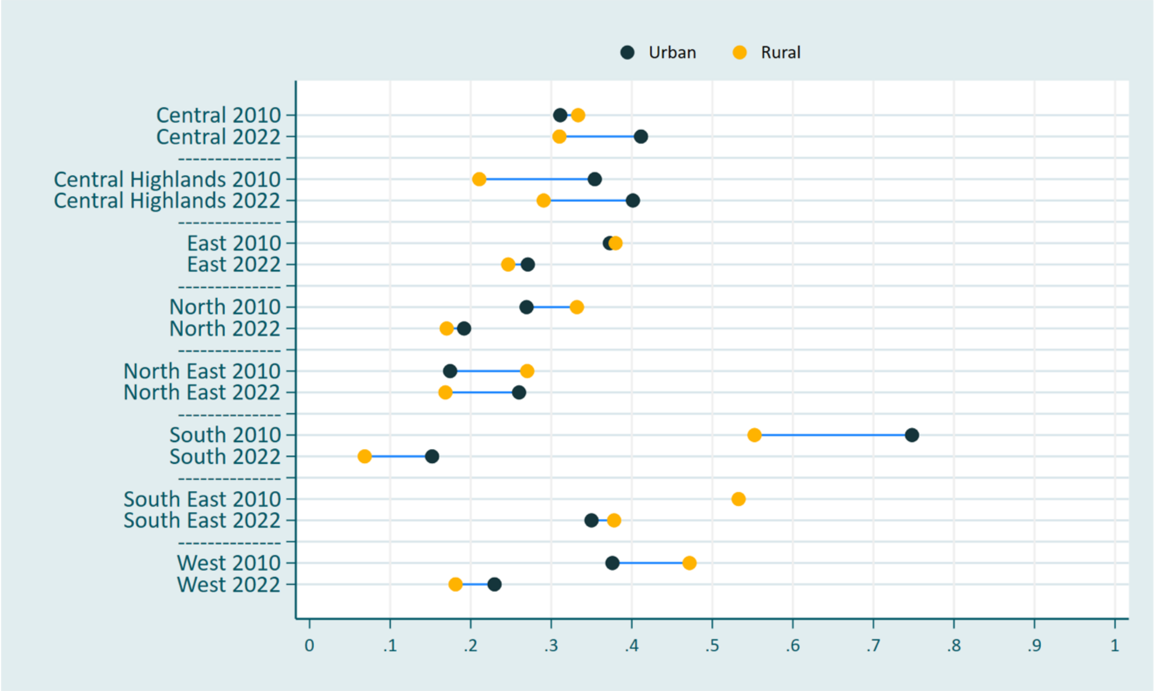

Supplemental Figure 2I - *Equiplot of oral rehydration therapy coverage within urban or rural residence and for each geographic region displayed in Supplemental Figure 1.*

Antenatal care visits

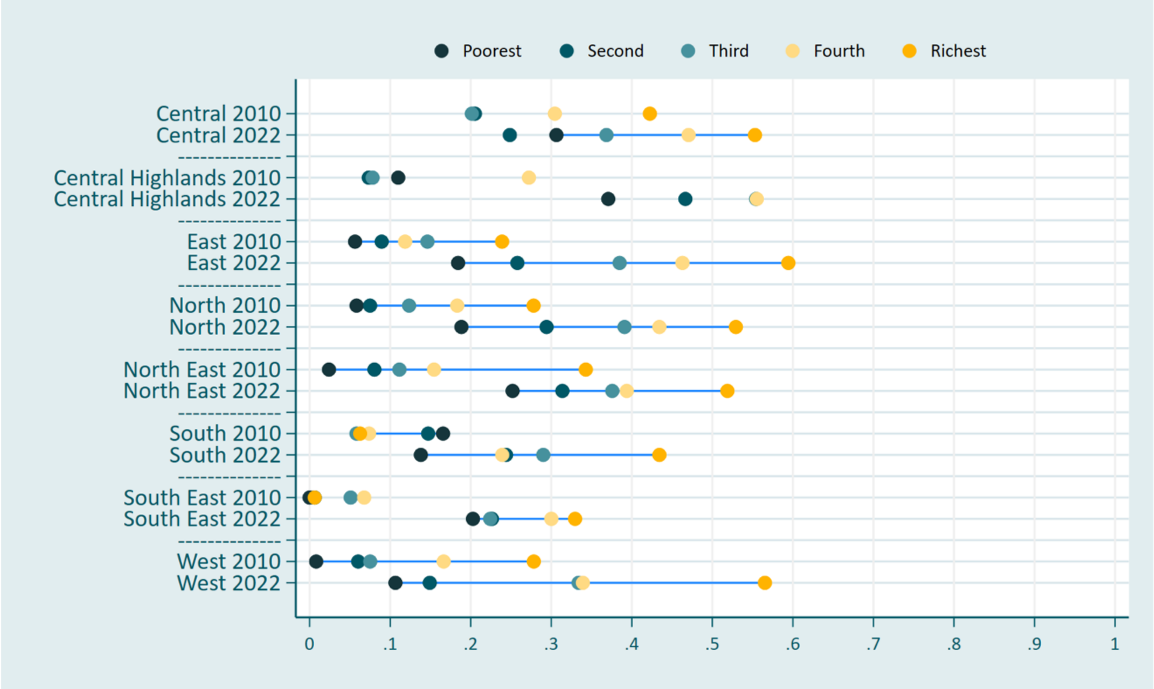

Supplemental Figure 2J - Equiplot of antenatal care visits within wealth quintiles and for each geographic region displayed in Supplemental Figure 1.

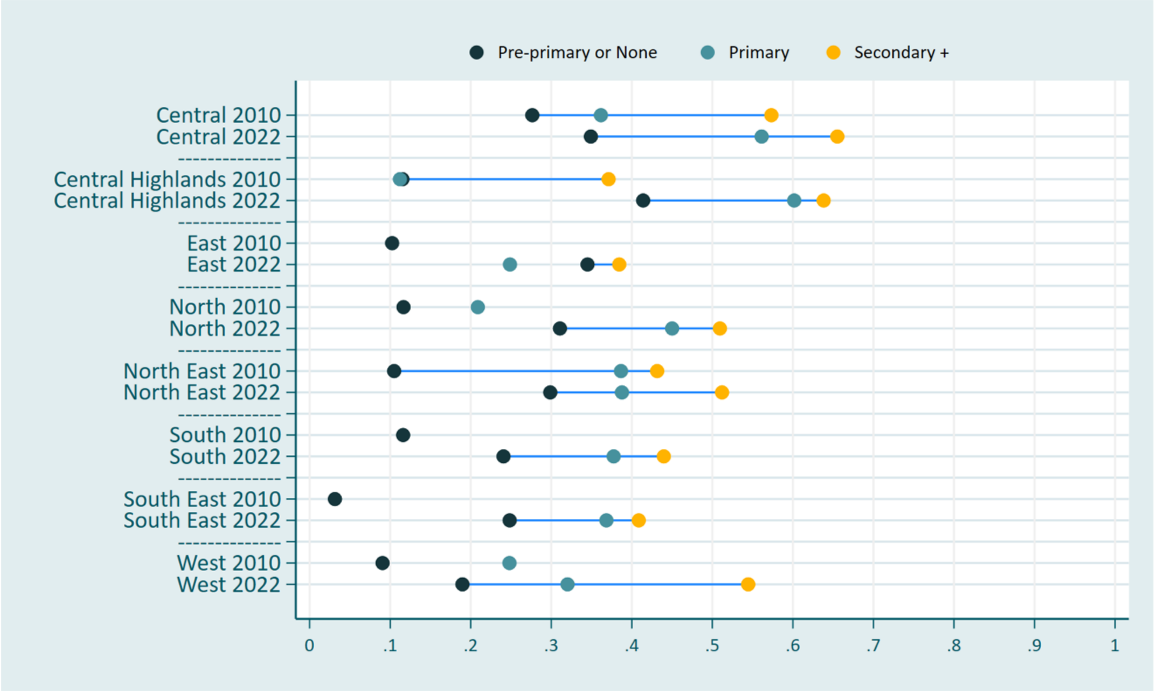

Supplemental Figure 2K - Equiplot of antenatal care visits within education groups and for each geographic region displayed in Supplemental Figure 1.

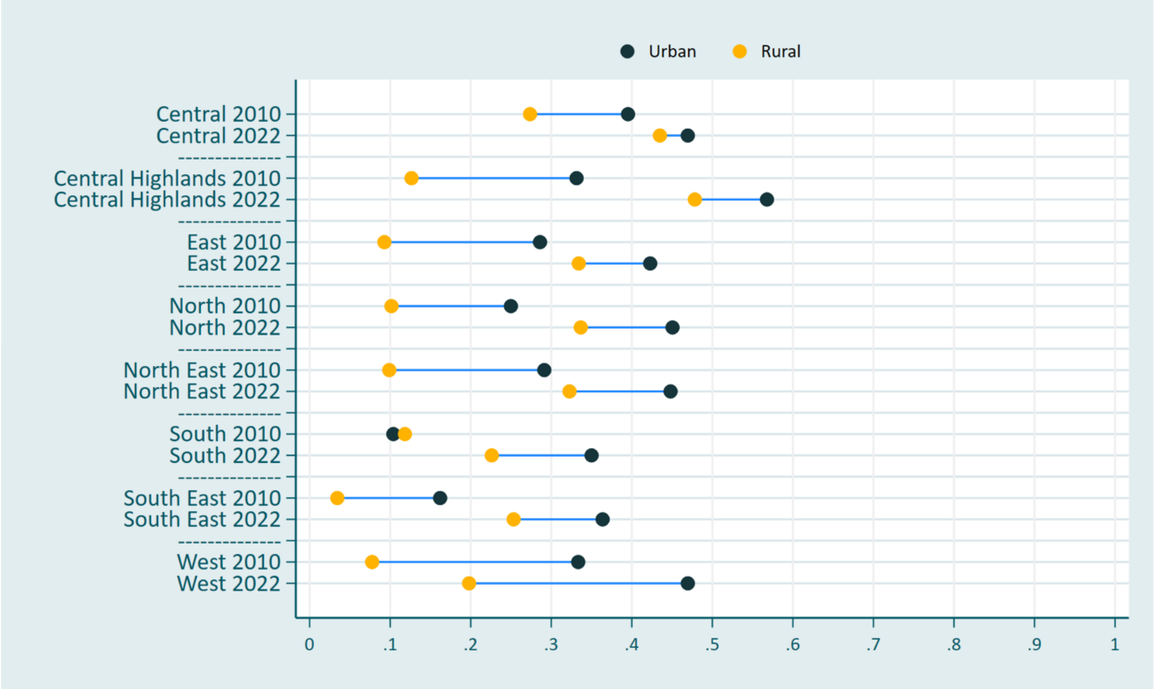

Supplemental Figure 2L - *Equiplot of antenatal care visits within urban or rural residence and for each geographic region displayed in Supplemental Figure 1.*

DPT3 immunization

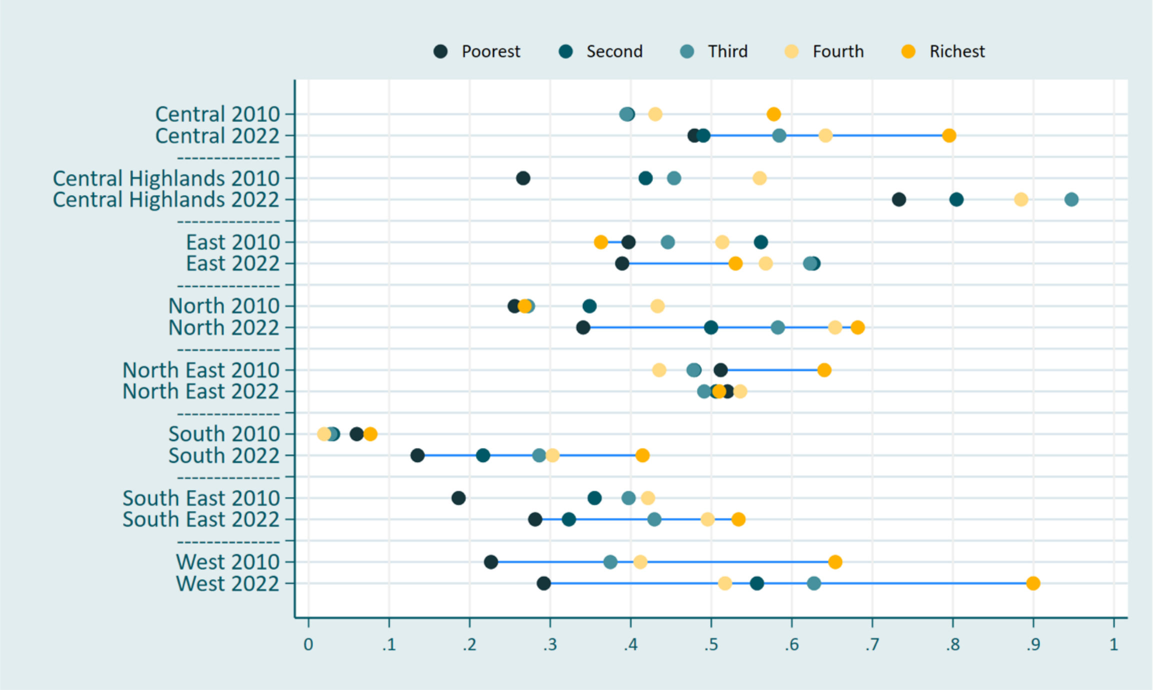

Supplemental Figure 2M- Equiplot of DPT3 immunization within wealth quintiles and for each geographic region displayed in Supplemental Figure 1.

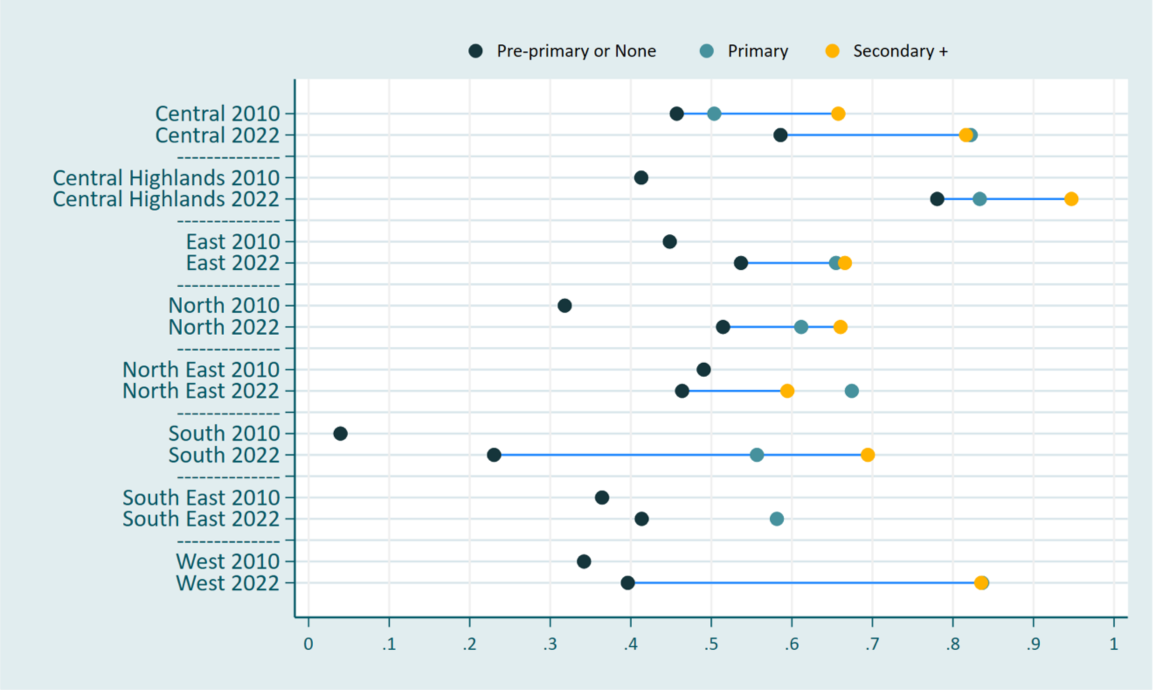

Supplemental Figure 2N - Equiplot of DPT3 immunization within education groups and for each geographic region displayed in Supplemental Figure 1.

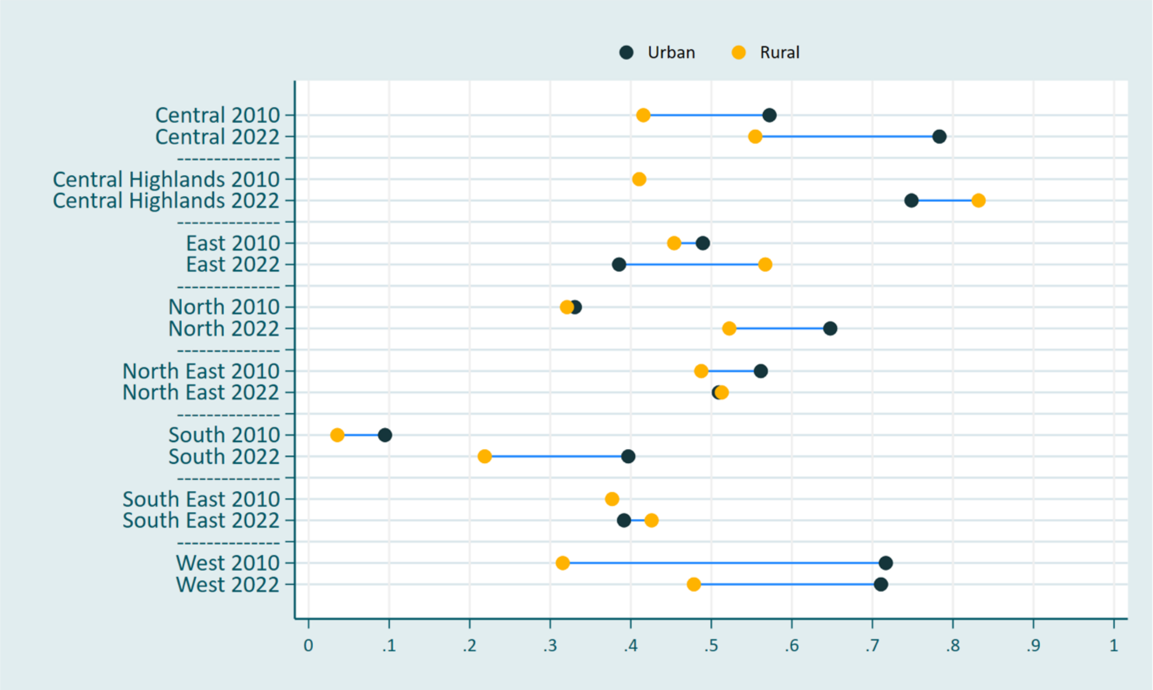

Supplemental Figure 2O - *Equiplot of DPT3 immunization within urban or rural residence and for each geographic region displayed in Supplemental Figure 1.*

Measles immunization

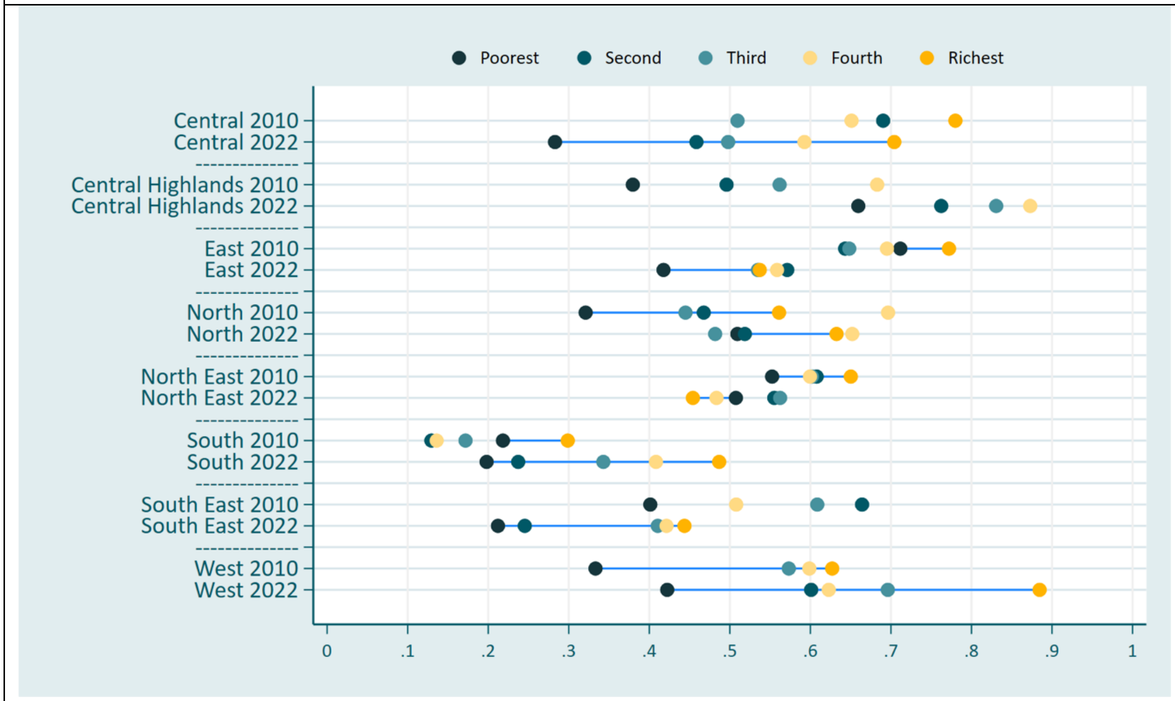

Supplemental Figure 2P - Equiplot of measles immunization within wealth quintiles and for each geographic region displayed in Supplemental Figure 1.

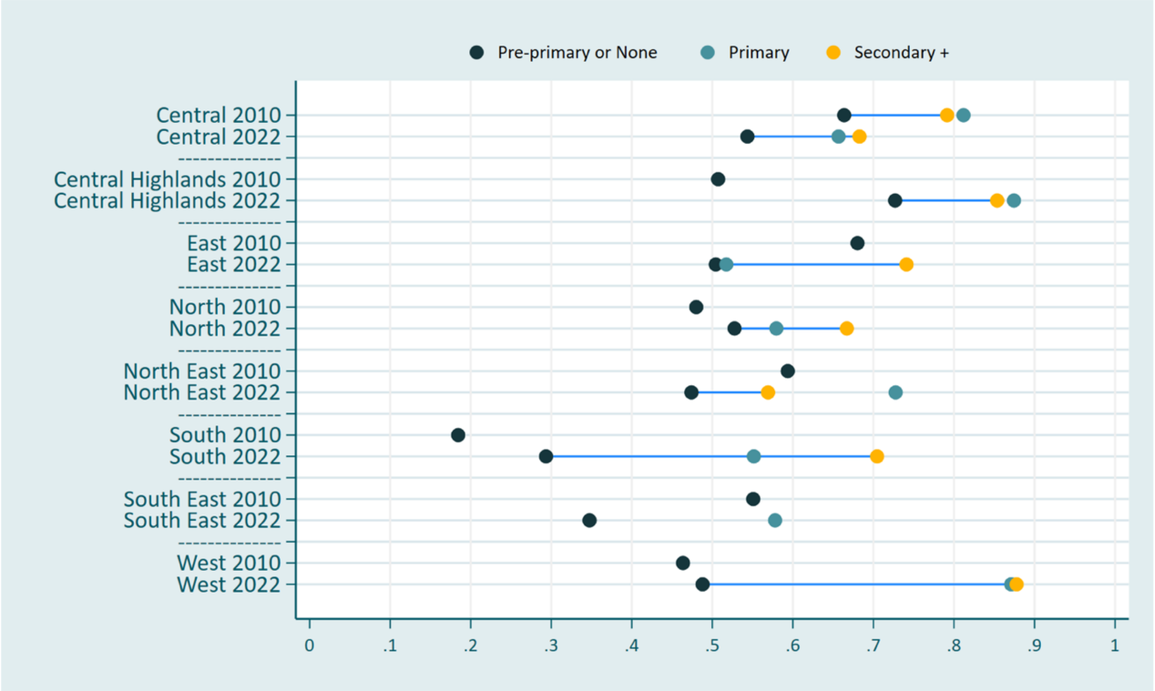

Supplemental Figure 2Q - Equiplot of measles immunization within education groups and for each geographic region displayed in Supplemental Figure 1.

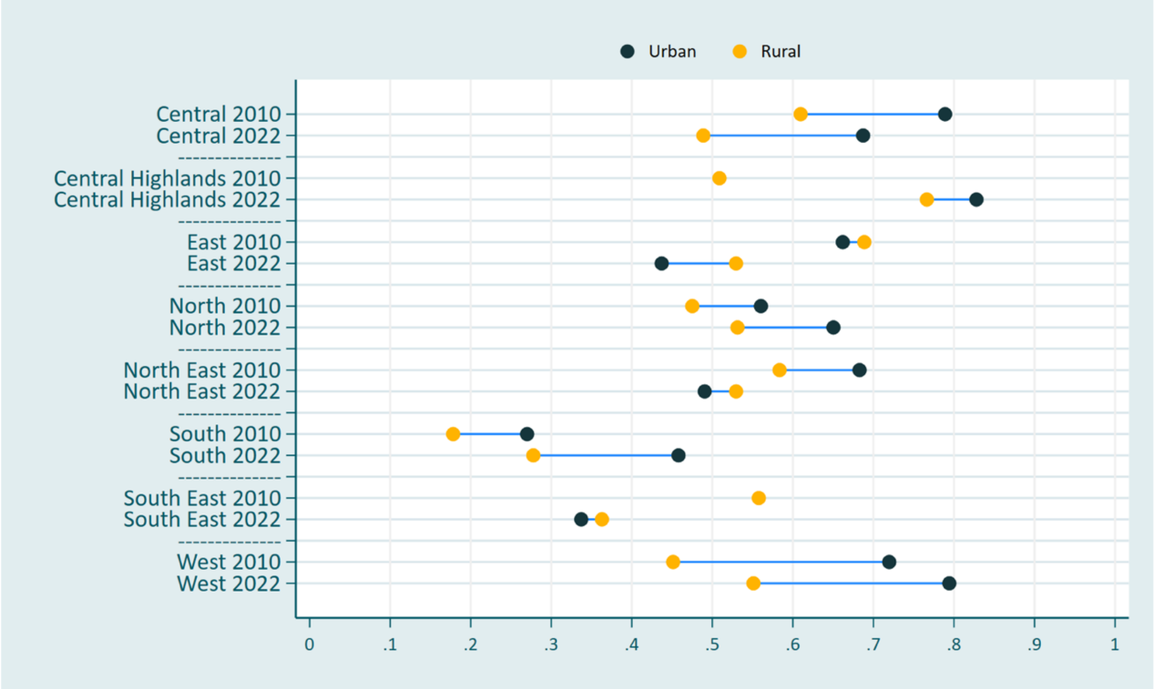

Supplemental Figure 2R - *Equiplot of measles immunization within urban or rural residence and for each geographic region displayed in Supplemental Figure 1.*

Treatment seeking for suspected pneumonia

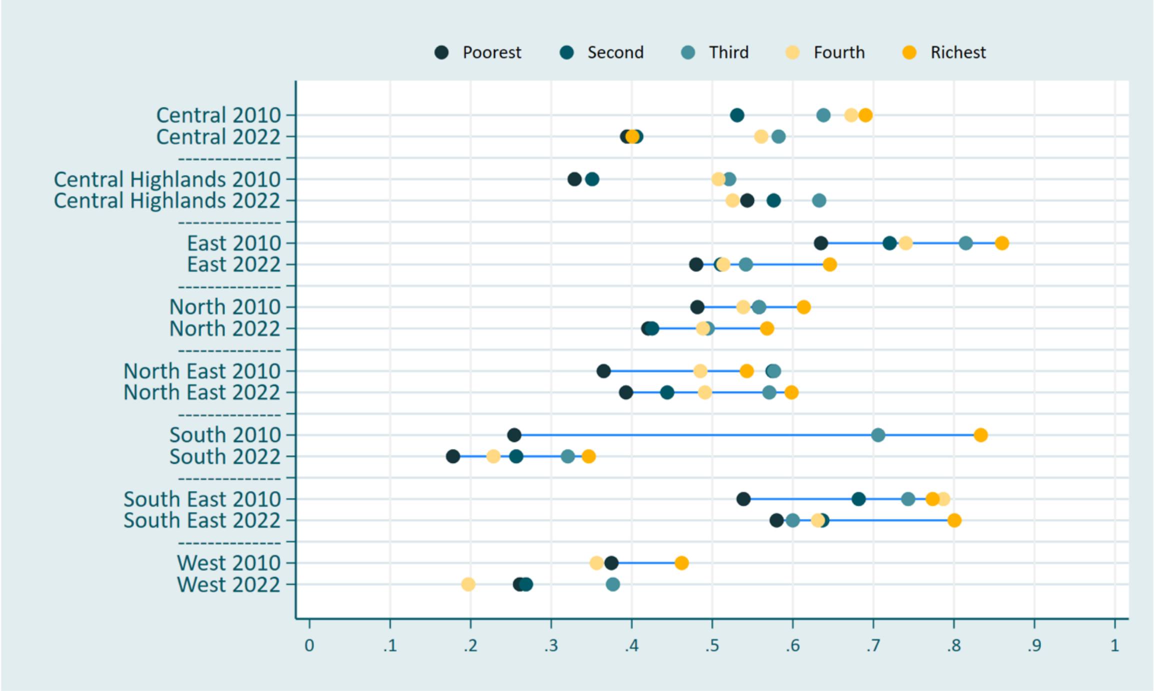

Supplemental Figure 2S - Equiplot of treatment for suspected pneumonia within wealth quintiles and for each geographic region displayed in Supplemental Figure 1.

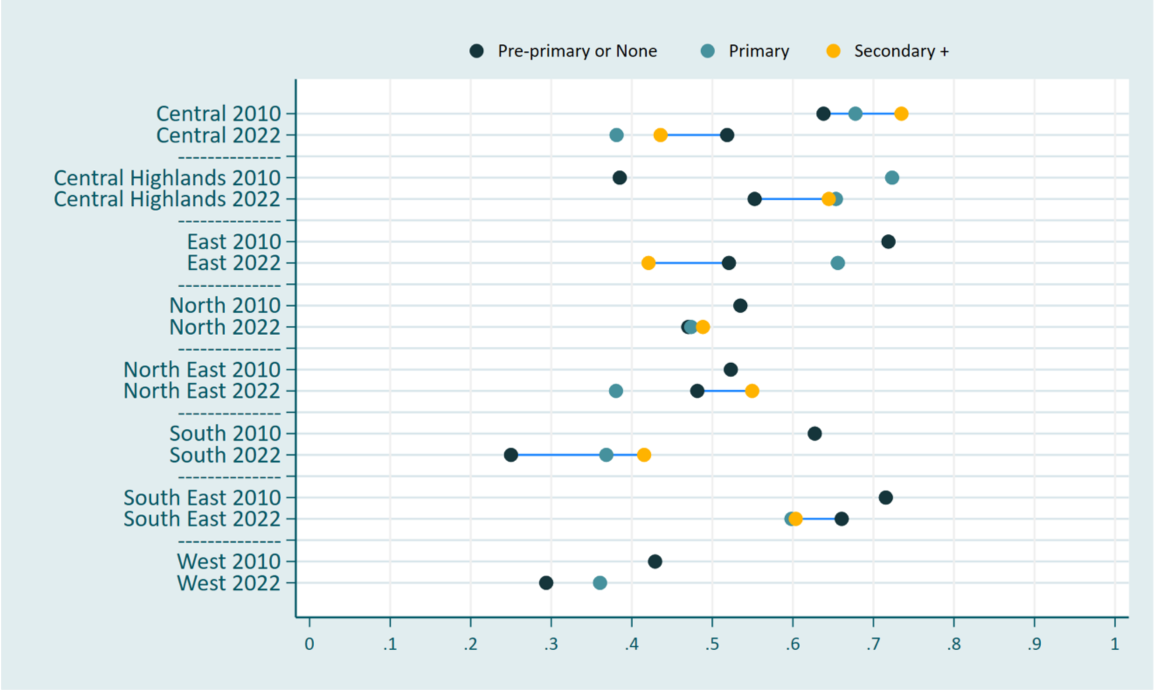

Supplemental Figure 2T - Equiplot of treatment for suspected pneumonia within education groups and for each geographic region displayed in Supplemental Figure 1.

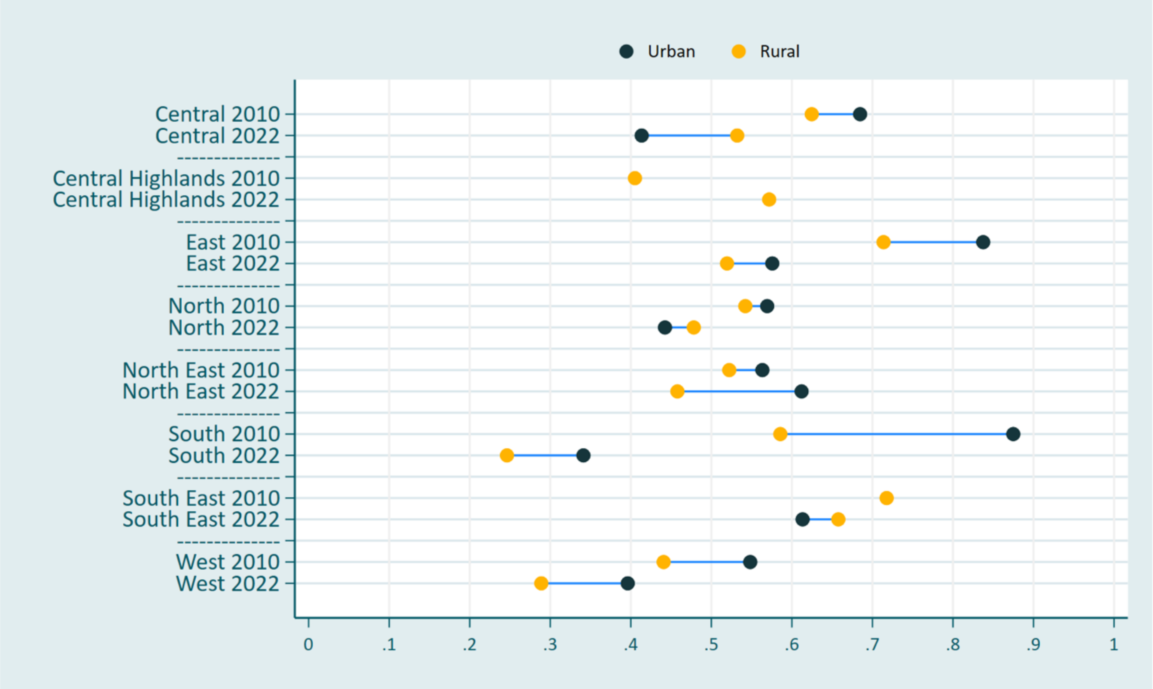

Supplemental Figure 2U - *Equiplot of treatment for suspected pneumonia within urban or rural residence and for each geographic region displayed in Supplemental Figure 1.*

Supplemental Figure 2 A-U – Equiplots for each of the seven indicators (antenatal care, skilled birth attendance, BCG, DPT3 and measles immunization, care-seeking for suspected pneumonia, and treatment of diarrhea with oral rehydration solution), by wealth, education, and urban/rural area, and over the geographic regions from Supplemental Figure 1

Antenatal care visits

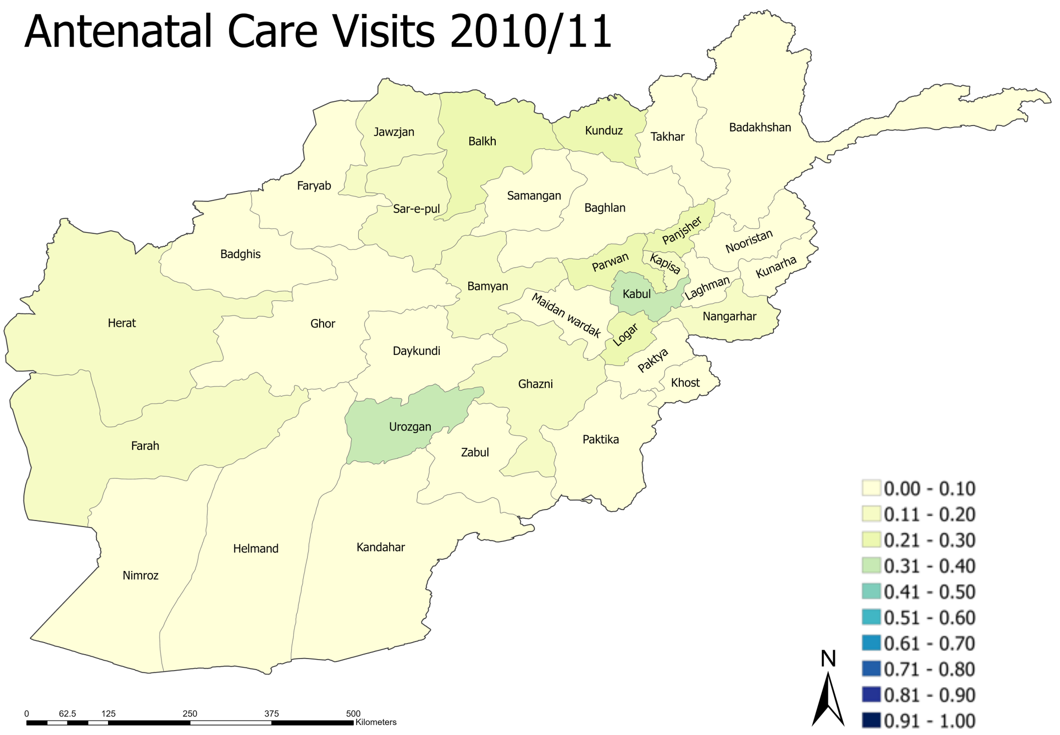

Supplemental Figure 3A - Mean coverage of antenatal care visits per province in the 2010/11 survey.

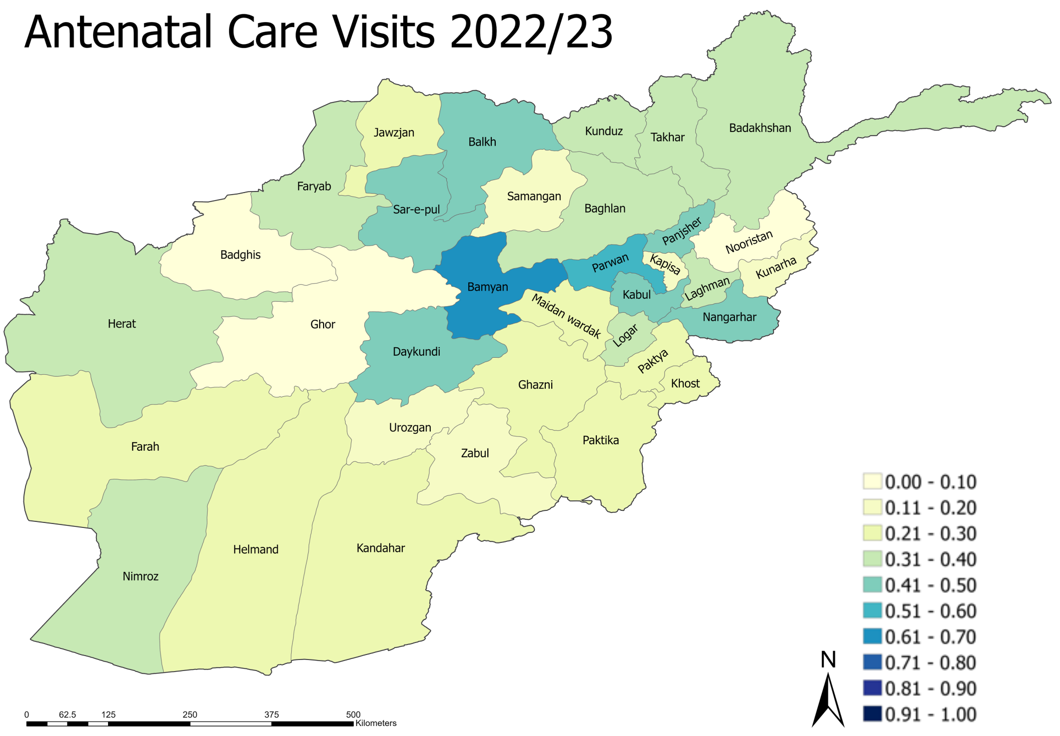

Supplemental Figure 3B - Mean coverage of antenatal care visits per province in the 2022/23 survey.

DPT3 immunization

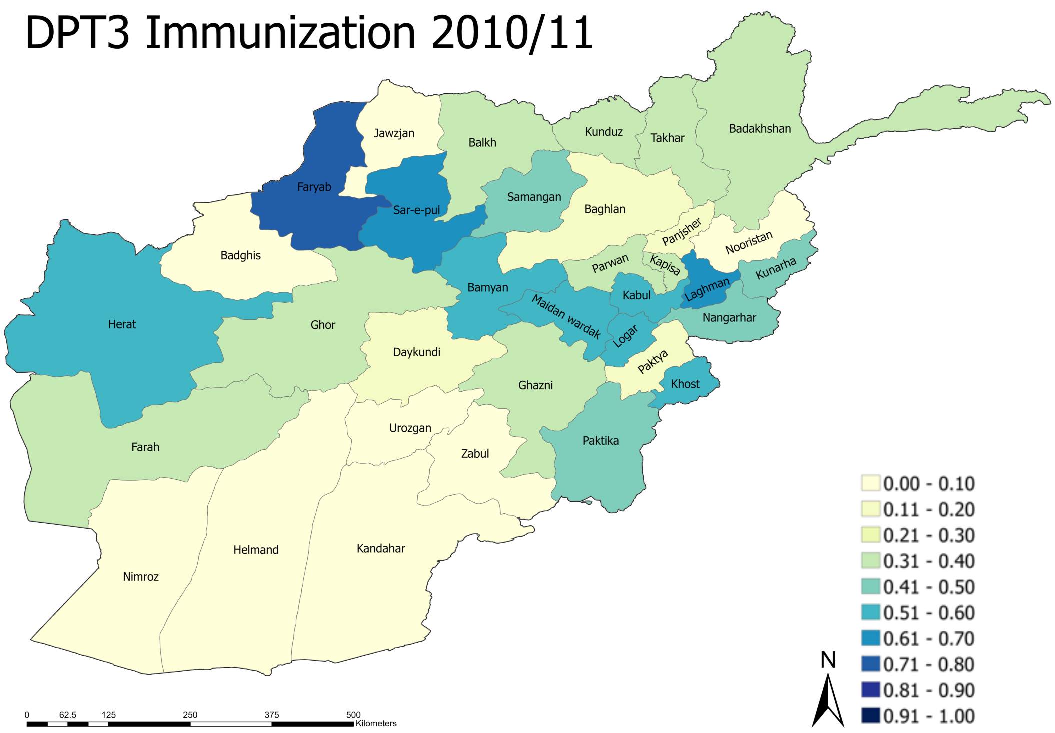

Supplemental Figure 3C - Mean coverage of DPT3 immunization per province in the 2010/11 survey.

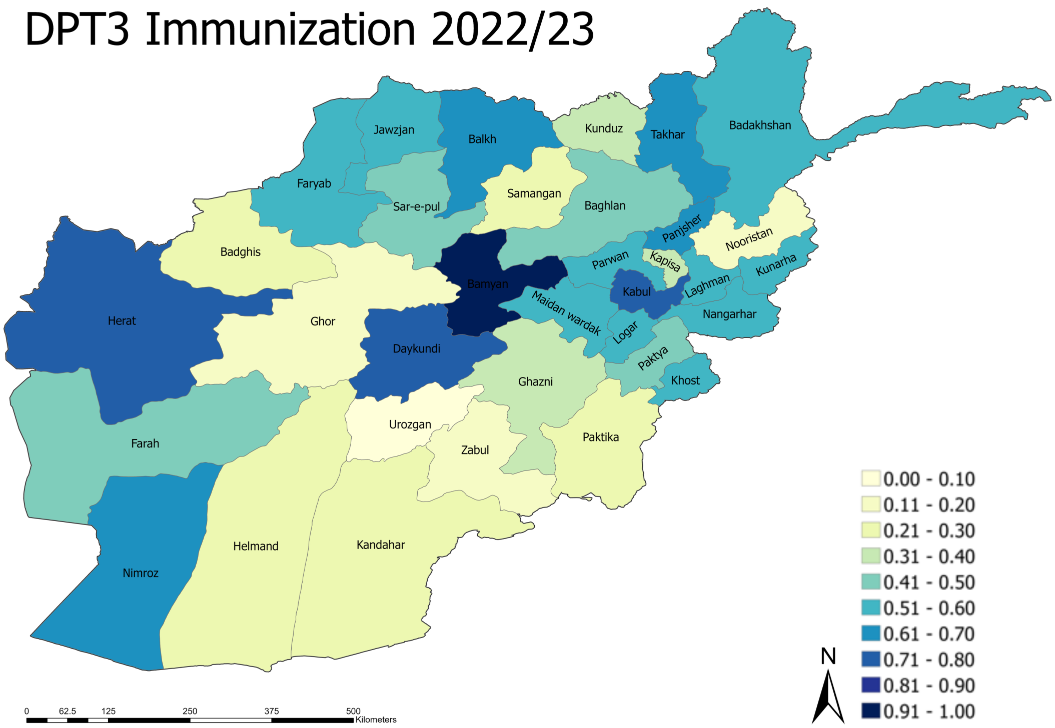

Supplemental Figure 3D - Mean coverage of DPT3 immunization per province in the 2022/23 survey.

Measles immunization

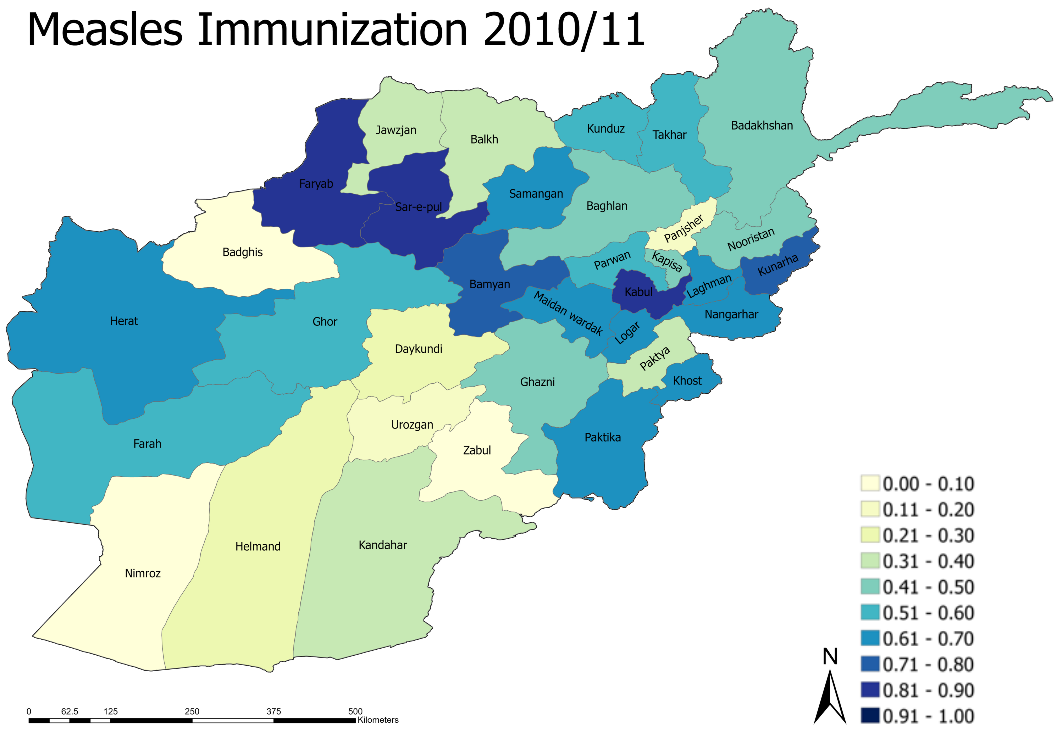

Supplemental Figure 3E - Mean coverage of measles immunization per province in the 2010/11 survey.

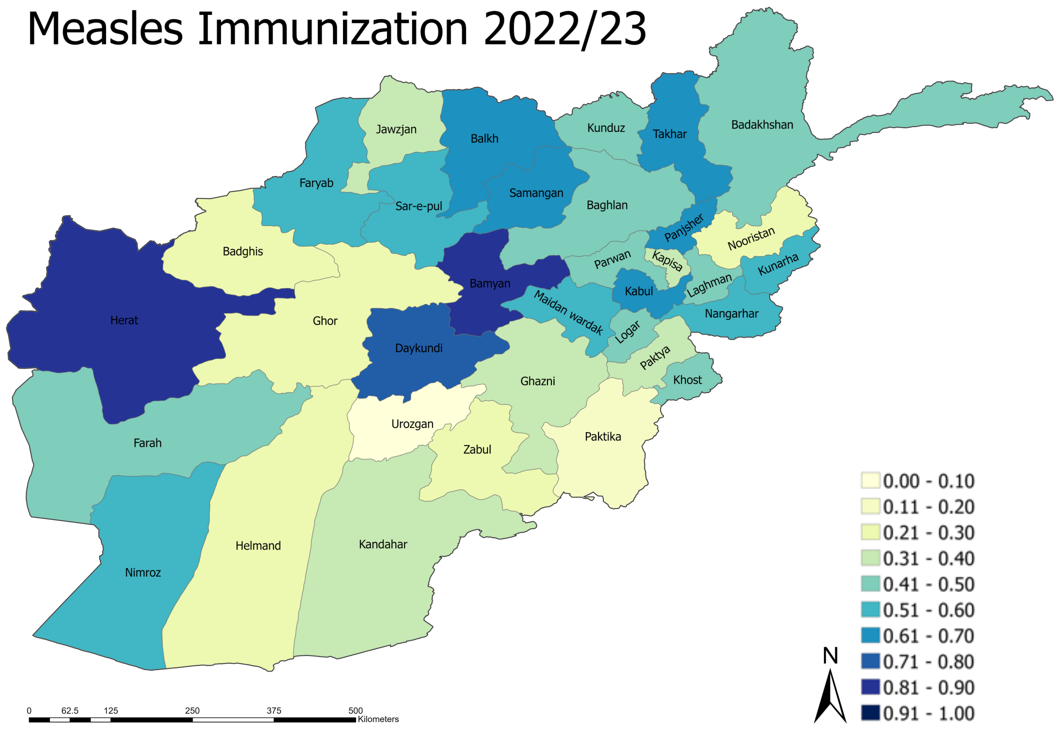

Supplemental Figure 3F - Mean coverage of measles immunization per province in the 2022/23 survey.

Treatment seeking for suspected pneumonia

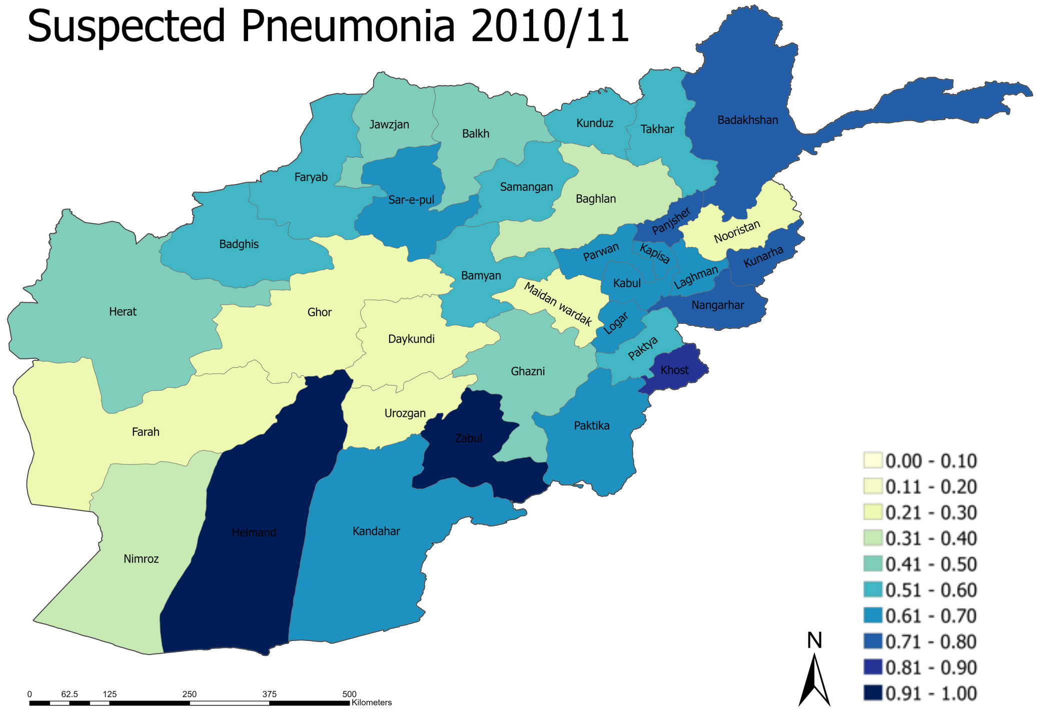

Supplemental Figure 3F - Mean coverage of treatment for suspected pneumonia per province in the 2010/11 survey.

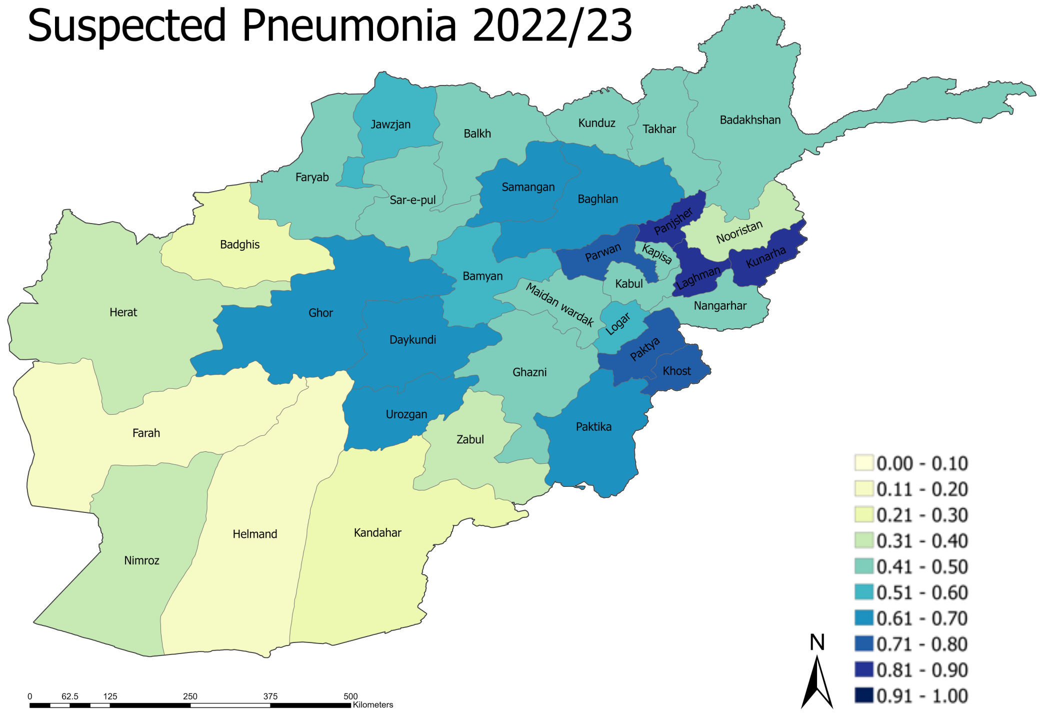

Supplemental Figure 3G - Mean coverage of treatment for suspected pneumonia per province in the 2022/23 survey.

Supplemental Figure 3 A-G -Spatial distribution of mean coverage rates for antenatal care, DPT3 and measles immunization, and suspected pneumonia for each of the 34 Afghan provinces. Exact values with corresponding 95% confidence interval in Supplemental Table 3

Composite Coverage Index

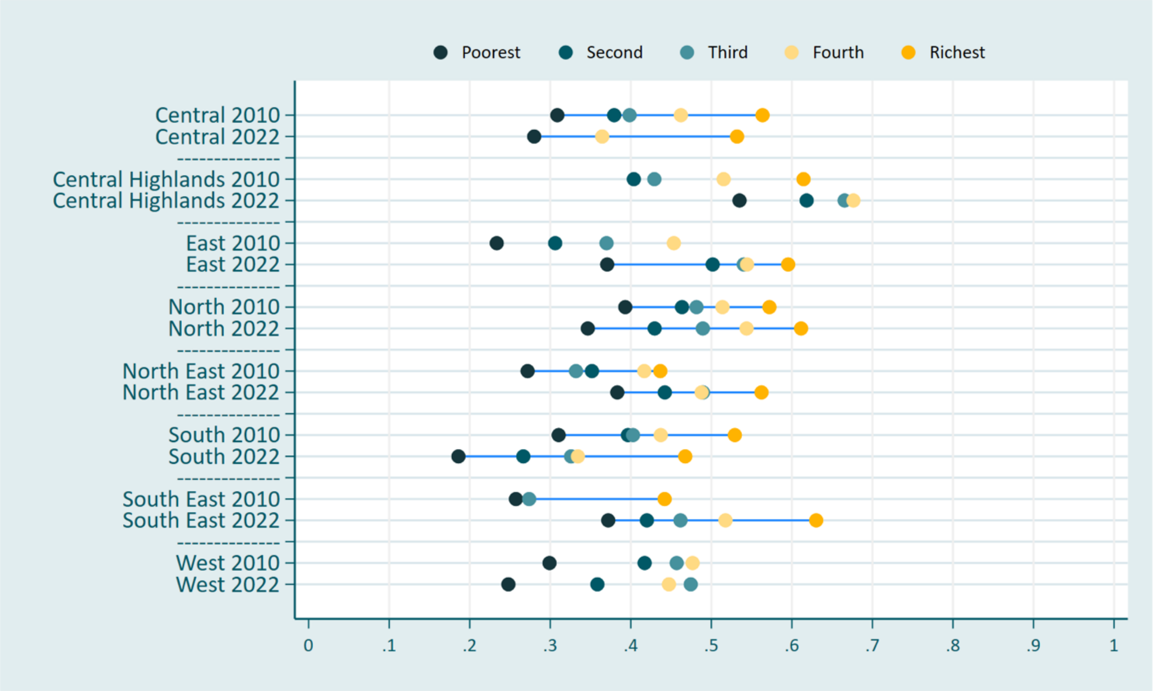

Supplemental Figure 4A - Equiplot of CCI values within wealth quintiles and for each geographic region displayed in Supplemental Figure 1

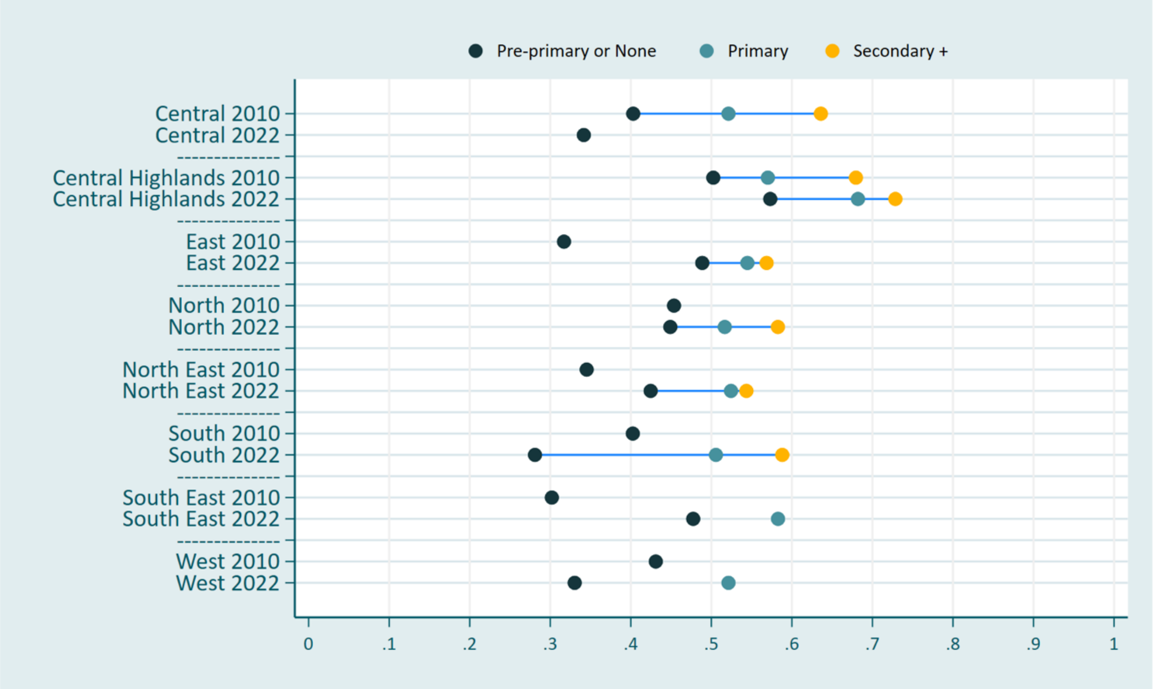

Supplemental Figure 4B - Equiplot of CCI values within education groups and for each geographic region displayed in Supplemental Figure 1

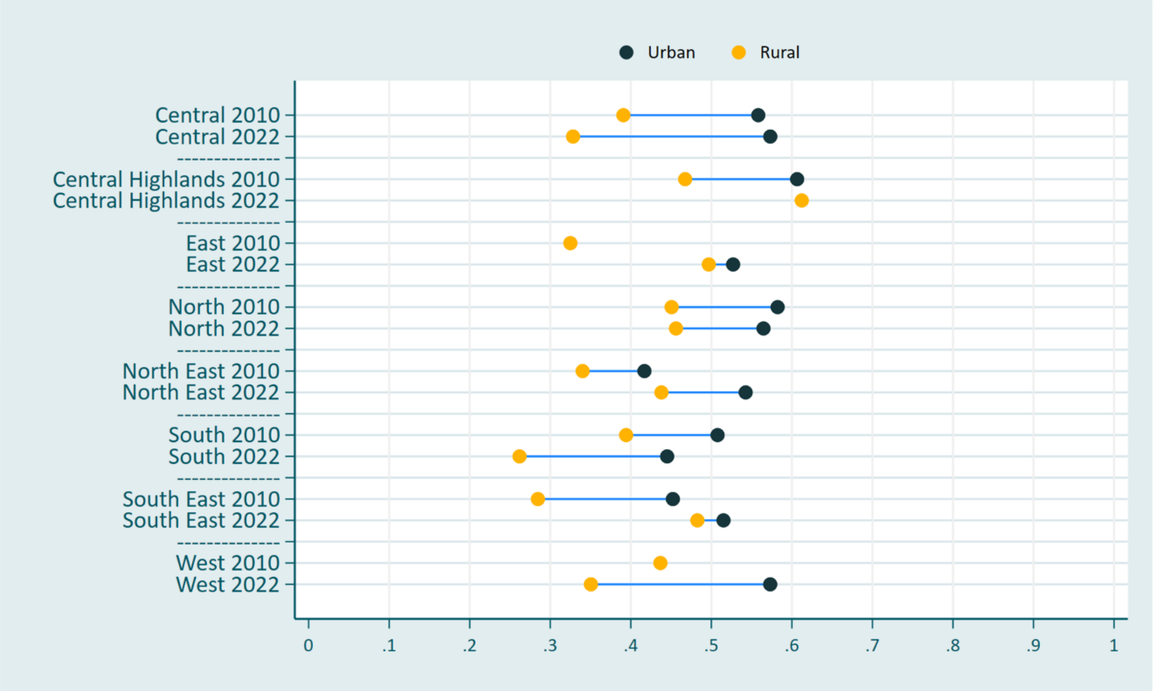

Supplemental Figure 4C - Equiplot of CCI values within urban or rural residence and for each geographic region displayed in Supplemental Figure 1

*Supplemental Figure 4A-C – Equiplots displaying regional (*Supplemental Figure 1*) inequalities by each of the three equity dimensions. A connecting line indicates the presence of all individual substrata connecting lowest and highest values.*

Province	ANC4 2010 (95% CI)	ANC4 2022 (95% CI)	SBA 2010 (95% CI)	SBA 2022 (95% CI)	BCG 2010 (95% CI)	BCG 2022 (95% CI)	DPT3 2010 (95% CI)	DPT3 2022 (95% CI)	MSL 2010 (95% CI)	MSL 2022 (95% CI)	ARI 2010 (95% CI)	ARI 2022 (95% CI)	ORS 2010 (95% CI)	ORS 2022 (95% CI)
**Kabul**	39.5% (34.3%, 45%)	49.8% (43.6%, 55.9%)	81.4% (75.5%, 86.2%)	94.2% (90.2%, 96.7%)	88.2% (83.3%, 91.9%)	89.9% (84.1%, 93.8%)	58.0% (51.1%, 64.5%)	77.2% (69.2%, 83.7%)	80.9% (75.4%, 85.4%)	66.9% (60.9%, 72.4%)	69.6% (63.2%, 75.4%)	42.2% (32.4%, 52.7%)	33.9% (29.0%, 39.3%)	40.8% (31.1%, 51.3%)
**Kapisa**	15.6% (9.5%, 24.6%)	16.4% (12.7%, 20.9%)	40.7% (31.5%, 50.6%)	74.4% (64.2%, 82.5%)	63.7% (37.7%, 83.5%)	52.1% (41.2%, 62.8%)	35.8% (17.0%, 60.4%)	36.2% (26.7%, 46.9%)	44.8% (21.9%, 70.2%)	32.2% (23.5%, 42.2%)	61.0% (53.0%, 68.5%)	44.3% (33.1%, 56.1%)	17.8% (12.8%, 24.1%)	46.1% (41.3%, 51.0%)
**Parwan**	30.4% (18.0%, 46.4%)	55.8% (45.8%, 65.3%)	43.5% (31.4%, 56.3%)	80.5% (65.7%, 89.8%)	65.9% (46.7%, 81.0%)	74.6% (62.2%, 84.0%)	35.6% (26.2%, 46.4%)	54.2% (42.1%, 65.9%)	58.5% (40.1%, 74.9%)	49.4% (37.8%, 61.1%)	63.6% (52.2%, 73.7%)	74.3% (65.5%, 81.4%)	33.4% (23.4%, 45.1%)	19.0% (14.6%, 24.4%)
**Maidan Wardak**	0.0% (no obs.)	29.7% (24.9%, 35.0%)	6.6% (1.2%, 29.2%)	74.0% (66.6%, 80.2%)	72.8% (37.4%, 92.3%)	70.2% (60.8%, 78.1%)	52.3% (17.8%, 84.8%)	57.6% (47.1%, 67.5%)	65.2% (31.7%, 88.4%)	57.2% (48.5%, 65.5%)	28.0% (16.3%, 43.7%)	42.6% (36.5%, 49.0%)	20.3% (12.3%, 31.7%)	30.2% (24.9%, 36.1%)
**Logar**	27.7% (12.2%, 51.5%)	39.5% (32.7%, 46.6%)	58.7% (38.6%, 76.3%)	81.5% (75.9%, 86.0%)	66.0% (13.5%, 96.1%)	75.5% (66.2%, 82.8%)	59.5% (11.0%, 94.6%)	51.7% (41.9%, 61.3%)	66.0% (13.5%, 96.1%)	41.7% (34.2%, 49.5%)	69.2% (54.9%, 80.6%)	55.7% (49.2%, 62.0%)	49.0% (25.3%, 73.3%)	31.6% (26.6%, 37.0%)
**Nangarhar**	12.1% (8.1%, 17.7%)	40.8% (33.0%, 49.0%)	46.8% (33.1%, 60.9%)	80.8% (73.9%, 86.2%)	77.9% (68.1%, 85.4%)	73.1% (65.5%, 79.5%)	46.1% (35.0%, 57.6%)	58.4% (49.2%, 67.1%)	68.6% (58.5%, 77.1%)	56.2% (46.8%, 65.1%)	76.3% (68.2%, 82.9%)	46.0% (41.6%, 50.4%)	33.0% (26.1%, 40.8%)	19.2% (13.2%, 26.9%)
**Laghman**	9.9% (4.6%, 19.9%)	32.3% (25.0%, 40.6%)	32.1% (18.8%, 49.1%)	57.5% (44.3%, 69.6%)	70.9% (47.0%, 87.0%)	65.4% (55.4%, 74.1%)	67.6% (38.2%, 87.6%)	56.2% (46.9%, 65.1%)	70.3% (43.4%, 88.0%)	48.0% (37.9%, 58.3%)	68.2% (52.9%, 80.3%)	81.4% (73.5%, 87.4%)	30.8% (21.9%, 41.4%)	35.0% (29.1%, 41.4%)
**Panjsher**	25.9% (11.9%, 47.5%)	46.1% (37.2%, 55.3%)	55.8% (26.2%, 81.7%)	77.0% (69.3%, 83.3%)	37.3% (30.2%, 45.1%)	70.5% (56.1%, 81.8%)	11.2% (3.9%, 28.4%)	61.1% (47.9%, 72.9%)	20.4% (3.4%, 65.2%)	61.2% (49.1%, 72.1%)	78.4% (27.8%, 97.2%)	83.9% (72.0%, 91.3%)	60.5% (43.6%, 75.3%)	24.9% (18.5%, 32.7%)
**Baghlan**	9.3% (4.1%, 19.8%)	30.6% (23.3%, 38.9%)	29.9% (20.8%, 41.0%)	60.4% (53.4%, 67.1%)	47.7% (33.3%, 62.6%)	65.3% (54.0%, 75.1%)	19.3% (12.2%, 29.3%)	49.4% (38.4%, 60.5%)	45.1% (30.4%, 60.8%)	43.9% (34.1%, 54.2%)	38.4% (27.2%, 51.1%)	63.3% (47.5%, 76.8%)	24.8% (16.5%, 35.6%)	13.3% (6.7%, 24.8%)
**Bamyan**	15.8% (10.3%, 23.5%)	63.2% (51.9%, 73.2%)	36.0% (29.4%, 43.1%)	82.9% (75.1%, 88.6%)	72.8% (60.8%, 82.1%)	98.6% (94.7%, 99.6%)	59.3% (47.9%, 69.8%)	92.6% (80.7%, 97.4%)	71.0% (61.0%, 79.4%)	88.7% (73.6%, 95.7%)	50.9% (38.8%, 62.9%)	54.9% (46.5%, 63.1%)	24.7% (18.5%, 32.2%)	35.3% (26.4%, 45.3%)
**Ghazni**	12.6% (5.6%, 25.7%)	24.7% (17.0%, 34.4%)	34.5% (25.4%, 44.9%)	72.8% (66.5%, 78.4%)	51.5% (34.3%, 68.4%)	46.1% (35.8%, 56.9%)	36.2% (19.7%, 56.7%)	38.5% (27.0%, 51.6%)	43.6% (27.8%, 60.9%)	39.3% (28.8%, 50.9%)	49.6% (32.0%, 67.3%)	42.6% (34.8%, 50.9%)	28.1% (20.1%, 37.8%)	20.0% (16.6%, 23.9%)
**Paktika**	1.0% (0.3%, 3.3%)	28.1% (20.4%, 37.2%)	28.0% (15.4%, 45.3%)	67.0% (55.4%, 76.9%)	68.6% (50.0%, 82.7%)	30.9% (24.8%, 37.8%)	44.8% (36.7%, 53.2%)	20.5% (14.4%, 28.5%)	67.3% (49.3%, 81.4%)	16.3% (11.6%, 22.5%)	64.6% (44.7%, 80.5%)	68.1% (58.1%, 76.7%)	75.9% (47.1%, 91.8%)	19.6% (13.6%, 27.3%)
**Paktya**	7.8% (3.8%, 15.0%)	21.5% (16.4%, 27.5%)	48.9% (27.4%, 70.8%)	70.3% (58.2%, 80.1%)	46.3% (17.4%, 77.8%)	49.2% (38.4%, 60.0%)	17.5% (6.6%, 39.2%)	43.7% (33.5%, 54.5%)	30.6% (12.9%, 56.7%)	37.9% (28.0%, 48.9%)	57.5% (28.5%, 82.2%)	73.7% (62.3%, 82.6%)	36.3% (19.5%, 57.3%)	28.0% (23.4%, 33.1%)
**Khost**	3.6% (0.8%, 14.4%)	29.1% (24.4%, 34.2%)	53.9% (41.5%, 65.8%)	84.3% (79.1%, 88.4%)	72.3% (41.7%, 90.5%)	73.2% (67.9%, 78.0%)	53.4% (24.1%, 80.5%)	56.8% (47.4%, 65.7%)	64.9% (38.1%, 84.7%)	42.2% (34.7%, 50.2%)	86.5% (78.1%, 92.0%)	78.6% (73.6%, 82.9%)	38.9% (24.4%, 55.7%)	67.5% (58.9%, 75.1%)
**Kunarha**	10.2% (3.8%, 24.6%)	15.9% (12.8%, 19.4%)	26.1% (16.4%, 39.0%)	76.9% (67.3%, 84.3%)	85.0% (69.4%, 93.4%)	66.7% (54.3%, 77.2%)	42.3% (27.4%, 58.8%)	55.1% (42.9%, 66.7%)	78.2% (67.1%, 86.4%)	52.4% (41.8%, 62.7%)	73.8% (57.0%, 85.7%)	81.9% (77.8%, 85.3%)	71.5% (58.4%, 81.7%)	33.8% (25.3%, 43.6%)
**Nooristan**	3.4% (0.6%, 17.9%)	6.7% (3.9%, 11.3%)	9.6% (2.8%, 28.4%)	9.5% (6.8%, 13.2%)	42.9% (32.3%, 54.3%)	25.6% (16.7%, 37.2%)	0.0% (no obs.)	12.2% (5.9%, 23.6%)	41.7% (30.7%, 53.7%)	20.7% (13.2%, 30.9%)	22.0% (9.3%, 43.7%)	39.1% (31.7%, 47.1%)	29.4% (12.8%, 54.0%)	38.2% (31.4%, 45.5%)
**Badakhshan**	6.0% (2.6%, 13.1%)	33.9% (27.8%, 40.5%)	10.5% (5.0%, 20.9%)	48.2% (38.7%, 57.9%)	56.8% (45.9%, 67.1%)	58.9% (48.2%, 68.9%)	36.0% (26.5%, 46.8%)	55.3% (44.4%, 65.7%)	41.0% (31.2%, 51.6%)	48.9% (40.5%, 57.3%)	73.2% (61.3%, 82.6%)	40.4% (28.3%, 53.9%)	62.6% (44.9%, 77.5%)	17.1% (10.1%, 27.4%)
**Takhar**	7.6% (4.8%, 11.9%)	37.2% (30.0%, 45.0%)	22.0% (14.3%, 32.2%)	74.5% (66.1%, 81.3%)	63.4% (52.6%, 73.0%)	84.6% (78.8%, 89.1%)	38.3% (27.8%, 50.1%)	66.2% (55.6%, 75.4%)	53.8% (40.4%, 66.6%)	69.3% (59.6%, 77.5%)	56.6% (46.5%, 66.2%)	47.1% (40.3%, 53.9%)	35.4% (25.8%, 46.4%)	26.0% (19.8%, 33.3%)
**Kunduz**	24.5% (19.1%, 30.7%)	36.5% (31.3%, 41.9%)	33.5% (25.5%, 42.6%)	69.5% (63.2%, 75.1%)	69.8% (52.3%, 82.9%)	50.6% (41.5%, 59.8%)	37.0% (27.7%, 47.3%)	33.2% (25.2%, 42.3%)	55.6% (41.9%, 68.5%)	43.3% (34.5%, 52.6%)	59.8% (44.6%, 73.2%)	44.6% (34.8%, 54.9%)	19.4% (12.8%, 28.3%)	10.9% (7.2%, 16.3%)
**Samangan**	5.5% (2.5%, 11.7%)	18.1% (12.4%, 25.7%)	34.2% (23.4%, 46.9%)	68.3% (62.1%, 74.0%)	73.8% (63.9%, 81.7%)	58.8% (49.2%, 67.8%)	43.0% (32.7%, 53.8%)	24.8% (16.3%, 35.7%)	68.9% (56.4%, 79.1%)	62.9% (51.5%, 73.1%)	54.5% (37.0%, 71.0%)	67.8% (55.0%, 78.5%)	14.2% (7.5%, 25.3%)	18.3% (11.7%, 27.5%)
**Balkh**	23.8% (15.3%, 35.1%)	44.5% (37.8%, 51.5%)	53.7% (41.4%, 65.6%)	70.0% (60.5%, 78.1%)	55.3% (42.1%, 67.8%)	74.7% (63.1%, 83.6%)	38.8% (26.6%, 52.5%)	66.0% (51.7%, 78.0%)	38.8% (27.8%, 51.0%)	61.2% (52.5%, 69.3%)	47.8% (33.1%, 62.9%)	42.7% (28.0%, 58.8%)	14.8% (9.4%, 22.5%)	16.9% (13.0%, 21.6%)
**Sar-E-Pul**	11.4% (4.8%, 24.7%)	40.3% (31.6%, 49.6%)	48.4% (37.7%, 59.2%)	76.4% (67.3%, 83.6%)	91.4% (83.5%, 95.7%)	74.7% (62.2%, 84.2%)	61.8% (49.9%, 72.5%)	49.9% (39.8%, 60.1%)	83.8% (69.9%, 92.0%)	57.5% (46.9%, 67.5%)	69.2% (60.3%, 76.8%)	42.7% (34.4%, 51.5%)	25.1% (17.7%, 34.2%)	19.6% (14.4%, 26.2%)

**Ghor**	1.1%<br>(0.2%, 6.6%)	8.6%<br>(5.0%, 14.2%)	9.8%<br>(5.9%, 15.9%)	21.0%<br>(14.5%, 29.4%)	50.9%<br>(33.7%, 67.9%)	31.9%<br>(19.4%, 47.7%)	30.9%<br>(17.3%, 48.9%)	10.4%<br>(5.5%, 18.7%)	51.9%<br>(30.7%, 72.4%)	27.1%<br>(18.4%, 38.1%)	24.1%<br>(13.4%, 39.4%)	60.1%<br>(48.6%, 70.6%)	65.3%<br>(44.0%, 81.8%)	32.1%<br>(26.0%, 38.8%)
**Daykundi**	9.6%<br>(5.0%, 17.6%)	43.4%<br>(33.8%, 53.5%)	15.0%<br>(8.8%, 24.4%)	65.0%<br>(52.2%, 76.0%)	33.6%<br>(19.9%, 50.8%)	84.6%<br>(76.3%, 90.4%)	16.5%<br>(9.1%, 28.0%)	79.1%<br>(66.1%, 88.0%)	27.6%<br>(17.5%, 40.7%)	73.5%<br>(61.5%, 82.8%)	29.0%<br>(22.1%, 36.9%)	60.2%<br>(51.4%, 68.5%)	17.2%<br>(12.9%, 22.6%)	28.1%<br>(22.5%, 34.6%)
**Urozgan**	32.1%<br>(18.7%, 49.3%)	10.1%<br>(6.2%, 16.2%)	35.8%<br>(16.2%, 61.6%)	36.7%<br>(29.7%, 44.3%)	70.7%<br>(52.9%, 83.7%)	9.6%<br>(4.7%, 18.5%)	8.3%<br>(3.3%, 19.3%)	3.4%<br>(1.3%, 8.3%)	11.9%<br>(5.5%, 24.0%)	8.6%<br>(3.8%, 18.1%)	23.9%<br>(7.3%, 55.5%)	60.0%<br>(47.8%, 71.1%)	69.5%<br>(53.5%, 81.9%)	28.2%<br>(22.8%, 34.3%)
**Zabul**	0.0%<br>(no obs.)	12.5%<br>(8.8%, 17.6%)	0.0%<br>(no obs.)	49.1%<br>(40.4%, 57.8%)	26.3%<br>(11.0%, 50.6%)	47.4%<br>(39.2%, 55.8%)	0.0%<br>(no obs.)	17.9%<br>(12.7%, 24.5%)	4.7%<br>(0.6%, 29.7%)	28.6%<br>(21.4%, 37.1%)	90.7%<br>(62.8%, 98.3%)	39.2%<br>(32.0%, 47.0%)	44.3%<br>(31.2%, 58.1%)	17.7%<br>(11.9%, 25.4%)
**Kandahar**	9.7%<br>(4.9%, 18.2%)	27.7%<br>(23.9%, 31.8%)	30.0%<br>(19.9%, 42.5%)	67.2%<br>(60.9%, 72.9%)	45.1%<br>(30.1%, 61.1%)	56.5%<br>(49.8%, 63.0%)	6.8%<br>(3.0%, 14.7%)	28.6%<br>(22.0%, 36.3%)	31.5%<br>(19.7%, 46.3%)	32.4%<br>(26.5%, 38.9%)	63.6%<br>(49.5%, 75.6%)	29.7%<br>(24.5%, 35.5%)	68.8%<br>(57.1%, 78.5%)	12.7%<br>(9.8%, 16.3%)
**Jawzjan**	14.3%<br>(7.7%, 25.1%)	29.4%<br>(24.6%, 34.6%)	44.5%<br>(30.6%, 59.3%)	80.3%<br>(72.7%, 86.1%)	44.5%<br>(19.3%, 72.9%)	62.4%<br>(50.5%, 72.9%)	7.0%<br>(2.3%, 19.4%)	50.1%<br>(40.2%, 60.0%)	34.3%<br>(16.4%, 58.0%)	39.1%<br>(29.9%, 49.1%)	45.2%<br>(20.3%, 72.8%)	55.3%<br>(44.2%, 65.8%)	14.3%<br>(5.5%, 32.3%)	33.0%<br>(26.0%, 40.7%)
**Faryab**	7.8%<br>(4.5%, 13.0%)	31.8%<br>(26.0%, 38.3%)	34.4%<br>(29.0%, 40.2%)	58.6%<br>(47.0%, 69.4%)	86.1%<br>(74.4%, 93.0%)	68.7%<br>(56.6%, 78.7%)	79.4%<br>(64.7%, 89.1%)	54.0%<br>(44.0%, 63.7%)	82.7%<br>(70.4%, 90.5%)	55.7%<br>(45.4%, 65.6%)	53.8%<br>(31.5%, 74.6%)	45.2%<br>(36.8%, 53.8%)	49.0%<br>(38.1%, 60.0%)	5.6%<br>(3.1%, 9.9%)
**Helmand**	6.7%<br>(1.8%, 22.0%)	26.4%<br>(21.7%, 31.7%)	16.6%<br>(9.1%, 28.4%)	52.4%<br>(45.7%, 59.1%)	21.3%<br>(7.3%, 48.1%)	30.4%<br>(23.0%, 39.0%)	3.9%<br>(1.1%, 13.0%)	21.8%<br>(14.0%, 32.3%)	21.8%<br>(8.4%, 46.0%)	29.5%<br>(20.2%, 40.8%)	91.4%<br>(55.3%, 98.9%)	19.0%<br>(13.1%, 26.6%)	62.8%<br>(42.6%, 79.3%)	3.9%<br>(2.6%, 5.7%)
**Badghis**	2.5%<br>(0.4%, 13.5%)	9.0%<br>(5.0%, 15.6%)	3.7%<br>(0.4%, 25.0%)	24.1%<br>(16.6%, 33.7%)	17.7%<br>(8.8%, 32.3%)	41.9%<br>(27.6%, 57.7%)	7.8%<br>(2.4%, 22.8%)	22.9%<br>(13.1%, 36.8%)	7.8%<br>(2.4%, 22.8%)	27.9%<br>(18.8%, 39.3%)	53.5%<br>(40.8%, 65.7%)	29.2%<br>(20.4%, 39.9%)	41.9%<br>(30.8%, 54.0%)	30.4%<br>(24.3%, 37.4%)
**Herat**	18.4%<br>(14.2%, 23.5%)	34.7%<br>(26.2%, 44.3%)	38.9%<br>(27.7%, 51.5%)	60.0%<br>(50.9%, 68.6%)	76.8%<br>(63.0%, 86.6%)	80.7%<br>(60.7%, 91.9%)	60.5%<br>(46.3%, 73.1%)	73.6%<br>(56.2%, 85.8%)	60.8%<br>(48.0%, 72.3%)	80.5%<br>(69.1%, 88.4%)	49.8%<br>(37.6%, 62.0%)	33.8%<br>(26.8%, 41.7%)	38.5%<br>(26.7%, 51.9%)	19.0%<br>(13.5%, 26.0%)
**Farah**	11.5%<br>(4.1%, 28.0%)	24.9%<br>(18.6%, 32.4%)	32.7%<br>(9.9%, 68.2%)	54.7%<br>(42.3%, 66.4%)	46.7%<br>(18.8%, 76.8%)	46.8%<br>(31.7%, 62.5%)	34.5%<br>(12.1%, 66.9%)	40.8%<br>(26.6%, 56.8%)	57.7%<br>(23.5%, 85.8%)	44.0%<br>(30.3%, 58.8%)	29.7%<br>(18.8%, 43.4%)	14.3%<br>(9.4%, 21.2%)	33.2%<br>(17.2%, 54.4%)	6.8%<br>(4.2%, 11.0%)
**Nimroz**	0.0%<br>(no obs.)	34.4%<br>(29.8%, 39.3%)	12.8%<br>(2.9%, 41.6%)	76.7%<br>(67.2%, 84.0%)	53.7%<br>(6.4%, 95.1%)	67.7%<br>(53.9%, 79.0%)	0.0%<br>(no obs.)	61.6%<br>(50.1%, 71.9%)	0.0%<br>(no obs.)	59.2%<br>(46.8%, 70.5%)	34.1%<br>(21.6%, 49.3%)	30.8%<br>(24.0%, 38.4%)	0.0%<br>(no obs.)	12.1%<br>(8.9%, 16.3%)

Supplemental Table 3 -Coverage as proportions for each indicator for each province and both survey time-points with corresponding weighted 95% confidence interval.

Province	CCI 2010/11	CCI 2022/23	Absolute Difference
Kabul	61.3%	63.8%	2.5 p.p.
Kapisa	37.7%	43.2%	5.5 p.p.
Parwan	44.8%	57.6%	12.8 p.p.
Maidan Wardak	29.3%	49.6%	20.4 p.p.
Logar	55.1%	53.1%	-2.0 p.p.
Nangarhar	47.9%	51.6%	3.7 p.p.
Laghman	46.6%	53.2%	6.6 p.p.
Panjsher	43.4%	59.8%	16.5 p.p.
Baghlan	27.9%	45.3%	17.4 p.p.
Bamyan	43.2%	70.4%	27.3 p.p.
Ghazni	34.9%	40.2%	5.3 p.p.
Paktika	47.2%	37.8%	-9.3 p.p.
Paktya	34.6%	46.8%	12.2 p.p.
Khost	50.7%	62.3%	11.6 p.p.
Kunarha	50.8%	53.9%	3.1 p.p.
Nooristan	17.7%	21.5%	3.8 p.p.
Badakhshan	39.6%	41.5%	1.9 p.p.
Takhar	36.4%	54.6%	18.2 p.p.
Kunduz	39.4%	40.3%	0.9 p.p.
Samangan	37.1%	43.0%	5.9 p.p.
Balkh	37.8%	51.4%	13.6 p.p.
Sar-e-Pul	50.5%	49.2%	-1.3 p.p.
Ghor	30.4%	26.9%	-3.4 p.p.
Daykundi	19.8%	59.2%	39.3 p.p.
Urozgan	35.2%	24.6%	-10.6 p.p.
Zabul	25.1%	29.1%	4.0 p.p.
Kandahar	36.3%	35.1%	-1.3 p.p.
Jawzjan	27.3%	49.8%	22.5 p.p.
Faryab	51.5%	42.9%	-8.6 p.p.
Helmand	33.8%	25.6%	-8.3 p.p.
Badghis	20.5%	25.1%	4.6 p.p.
Herat	45.8%	50.3%	4.5 p.p.
Farah	32.2%	31.1%	-1.0 p.p.
Nimroz	12.3%	46.5%	34.2 p.p.

Supplemental Table 4 - Mean CCI for each province in both surveys including crude percentage point difference between 2022/23 and 2010/11

SUPPLEMENTAL REFERENCES

1 Afghanistan Multiple Indicator Cluster Survey - SURVEY FINDINGS REPORT. 2010.

2 Countdown 2030 – Maternal, Newborn & Child Health Data – Countdown to 2030. https://www.countdown2030.org/ (accessed 8 July 2024)

3 Victora CG, Requejo JH, Barros AJD, *et al.* Countdown to 2015: A decade of tracking progress for maternal, newborn, and child survival. The Lancet. 2016;387:2049–59.

4 Afghanistan MICS Multiple Indicator Cluster Survey Afghanistan MICS 2022-23 Multiple Indicator Cluster Survey 2022-23 Summary Findings Report. 2023.
